# ZIC2 affects oral squamous cell carcinoma stemness by regulating glycerophosphocholine metabolism via LYPLA2

**DOI:** 10.1038/s41419-026-08483-w

**Published:** 2026-03-19

**Authors:** Siyi Li, Xingyue Ma, Yuantao Li, Ming Yan, Longwei Hu, Ran Li, Yong Chen, Haiyang Li, Bowen Wang, Jianping Liu, Xiaoyan Zhang, Shuang Mei, Xiangjun Li

**Affiliations:** 1 Department of Oral & Maxillofacial-Head & Neck Oncology, Shanghai Ninth People’s Hospital, Shanghai Jiao Tong University School of Medicine, College of Stomatology, Shanghai Jiao Tong University, National Center for Stomatology, National Clinical Research Center for Oral Diseases, Shanghai Key Laboratory of Stomatology, Shanghai, China; 2 Department of Oral and Maxillofacial Surgery, School of Stomatology, Hebei Medical University, Hebei Technology Innovation Center of Oral Health, Key Laboratory of Stomatology and Clinical Research Centre for Oral Diseases, Shijiazhuang, Hebei Province China; 3 Shanghai Research Institute of Stomatology; Shanghai Center of Head and Neck Oncology Clinical and Translational Science, Shanghai, China; 4 Department of Oral and Maxillofacial Surgery, Cangzhou Central Hospital, Cangzhou, China

**Keywords:** Cancer metabolism, Cancer stem cells

## Abstract

Oral squamous cell carcinoma (OSCC) is a common malignant tumor of the head and neck. Early-stage OSCC is primarily treated using surgery; advanced-stage OSCC is managed using a multidisciplinary approach, including surgery combined with adjuvant radiotherapy and chemotherapy. However, tumor recurrence and metastasis remain major challenges, with a 5-year survival rate of ~50%. Dysregulation of transcription factors is associated with the pathogenesis of various cancers. This study focused on the role of ZIC2, a member of the zinc finger protein family, in OSCC. ZIC2 was identified as a prognostically relevant transcription factor in OSCC through bioinformatic analysis, showing high expression in OSCC and association with poor prognosis in patients. In vitro and in vivo, ZIC2 knockdown inhibited the proliferation, migration, invasion, and spheroid formation ability of OSCC cells and restored their sensitivity to chemotherapeutic drugs; overexpression of ZIC2 showed the opposite effect. RNA-seq and targeted metabolomics analyses revealed that in OSCC cells with zic2 knockdown, the expression of glycerophosphocholine (GPC) and the key rate-limiting enzyme LYPLA2 was decreased. LYPLA2 overexpression rescued the effects of ZIC2 knockdown on the proliferation, migration, and invasion of OSCC cells. GPC increased the stemness of OSCC tumor cells; ZIC2-regulated GPC metabolism through LYPLA2, inducing changes in the expression of the cancer stem cell markers Nanog and OCT4. In conclusion, we identified ZIC2 as an OSCC stemness-related gene, a potential therapeutic target for OSCC, providing new insights for treating OSCC.

## Introduction

Oral squamous cell carcinoma (OSCC) is a common malignant tumor of the head and neck region [1–3]. Its incidence is influenced by multiple factors, including tobacco use, chewing betel nut, human papillomavirus infection and genetic factors [4]. Long-term exposure to these factors can result in genetic mutations, epigenetic modifications, and dysregulation of the tumor microenvironment, leading to tumor initiation and progression [4]. OSCC treatment usually considers the clinical pathological stage, life expectancy, and quality of life. Surgery is the mainstay of treatment for early-stage OSCC, whereas advanced-stage OSCC is managed using a multidisciplinary approach, including surgery combined with adjuvant radiotherapy and chemotherapy. However, this cannot prevent tumor recurrence and metastasis [5]. Cisplatin and fluorouracil combined with pembrolizumab is the first-line treatment for OSCC [6]; nevertheless, two-thirds of patients with advanced OSCC show poor responses or develop resistance to this treatment. Similarly, the emergence of radiotherapy tolerance can significantly reduce radiotherapy efficacy [7]. Despite progress in diagnosis and treatment strategies, the 5-year survival rate of OSCC remains ~50%, necessitating the development of more effective treatment strategies [8].

Dysregulation and abnormal expression of transcription factors are closely associated with the pathogenesis of various human cancers [9]. Zinc finger proteins are highly conserved, key transcription factors in multiple cellular and biological processes [10]. ZIC2, a zinc finger transcription factor, is abnormally expressed in various tumors, such as ovarian cancer [10], liver cancer [11], renal clear cell carcinoma [12], nasopharyngeal carcinoma [13], and breast cancer [14], and is associated with poor prognosis in patients. Cancer stem cells (CSCs) are a subset of cancer cells with stem cell-like properties, characterized by self-renewal and differentiation capabilities. They have been implicated in tumor dormancy, treatment resistance, and distant metastasis, ultimately leading to treatment failure and tumor recurrence [15]. CSCs are found in various cancers, including head and neck, ovarian, pancreatic, colon, and breast cancers; their strong regenerative ability promotes tumor metastasis and infiltration [16]. CSCs play crucial roles in the growth, spread, and recurrence of oral cancer. The expressions of CSC markers, such as OCT4, SOX2, Nanog, nestin, CK19, Bmi-1, CD117, CD44, and CD133, are elevated in oral cancer, whereas those of involucrin and CK-13 are decreased [17]. Although some studies have reported that ZIC2 regulates tumor cell stemness [18, 19]. However, the involvement of ZIC2 in CSCs in oral cancer has not been reported. This study aimed to investigate this role.

Lipids store energy in the body and serve as signaling molecules. They are closely associated with the generation and colonization of tumor cells [20]. Tumor cells undergo different stages of lipid-related metabolic and structural adaptations, overcome cell death mechanisms, and promote lipid catabolism and anabolism to acquire energy and protect against oxidative stress [20]. The main lipid types include phospholipids, sphingolipids, triglycerides, fatty acids, and sterols. In the catabolic pathway, phosphatidylcholine is decomposed into fatty acids and glycerophosphocholine (GPC), which is further broken down into free choline and glycerol-3-phosphate, thereby participating in the choline metabolic cycle [21]. Enzymes related to the GPC metabolic pathway, such as cPLA2, lyso-PLA1, GDPD5, and GDPD6, are overexpressed in cancer and associated with tumor cell proliferation, migration, and invasion. Some tumour-related signaling pathways and transcription factors can affect GPC levels in cancer cells by regulating the expression of GPC-related enzymes, including cPLA2, lyso-PLA1, GDPD5, and GDPD6 [22]. High ZIC2 expression in OSCC was first reported in 2010 [23]. However, its specific molecular mechanism in OSCC remains unclear. In this study, we found that the high ZIC2 expression in OSCC was associated with poor patient prognosis. Lipid metabolism-related pathways were enriched in OSCC cell lines with ZIC2 knockdown. Targeted metabolomics revealed reduced GPC levels in ZIC2 knockdown OSCC cells. Exogenous GPC supplementation enhanced OSCC tumor stemness, suggesting that ZIC2 may promote OSCC development by regulating GPC metabolism. This study explores the role and mechanism of the prognostically relevant transcription factor ZIC2 in OSCC, providing a basis for its potential as a therapeutic target.

## Materials and methods

### Data collection

Original OSCC data were retrieved from The Cancer Genome Atlas (TCGA) database (https://portal.gdc.cancer.gov/) in April 2023, excluding non-oral head and neck squamous cell carcinoma sites, such as the hypopharynx, larynx, lip, tonsil, and oropharynx. In total, 32 OSCC and 32 paired tissue samples were selected, and corresponding RNA-seq transcriptome and clinical data were obtained. Eighty samples were collected from Gene Expression Omnibus (GEO) (https://www.ncbi.nlm.nih.gov/geo/) GSE37991 dataset, including 40 OSCC samples and 40 paired control samples.

### Identification of differentially expressed genes

Transcriptome data of TCGA-OSCC were analyzed, and differentially expressed genes with screening criteria of |log2FC | ≥ 1.5 and *P*adj < 0.05 were obtained; upregulated differentially expressed genes in TCGA-OSCC (Supplementary table 1) were identified. The TCGA-OSCC prognosis-related dataset was selected from the Xiantao Academic Database (Supplementary table 2), and information on transcription factors from AnimalTFDB (Animal Transcription Factor DataBase) (Supplementary table 3) was used. The intersection of these three was used to determine prognosis-related transcription factors in OSCC.

### Cell culture

The OSCC cell lines SCC-9 and CAL-27 were purchased from Procell Biology (Wuhan, China). The HN6 cell line was obtained from the Shanghai Key Laboratory of Stomatology, and human normal oral keratinocytes (HOK) were obtained from Otwo Biotech (Guangzhou, China). All cell lines were authenticated by STR profiling and treated with Beyotime Mycoplasma Remover (Cat. No.: C0288S) prior to experiments to ensure the absence of mycoplasma contamination. SCC-9 cells were cultured in DMEM/F12 supplemented with 10% fetal bovine serum (FBS; Gibco) and 1% penicillin–streptomycin solution 100× (Solarbio). HOK, CAL-27, and HN6 cells were cultured in DMEM containing 10% FBS (Gibco) and 1% 100× penicillin–streptomycin solution (Solarbio), and incubated at 37 °C in an atmosphere with 5% CO_2_.

### Tissue collection

Cancerous and adjacent tissues from nine patients with OSCC were collected at Cangzhou Central Hospital and stored at –80 °C for western blot experiments. Fifteen pairs of lesion tissues from patients with OSCC at Shanghai Ninth People’s Hospital, and seven pairs of gingival tissues from wisdom tooth extractions at Hebei Medical University Stomatological Hospital were collected as normal controls for immunohistochemical analysis. The inclusion criteria for OSCC patients were as follows: postoperative pathological confirmation of OSCC, no prior treatment before surgery, and availability of complete pathological and clinical follow-up data. The exclusion criterion was a pathological diagnosis other than OSCC. All patients provided informed consent, and the study adhered to the tenets outlined in the Declaration of Helsinki. Sample information can be found in Supplementary Materials 1 and 2.

### RNA extraction and real-time PCR

Total RNA was extracted from the cells using the R0026 kit (Beyotime, China) and reverse transcribed into complementary DNA (cDNA) using the PrimeScript RT kit (Takara, Japan). Real-time fluorescence quantitative PCR was performed using the SYBR Premix Ex Taq kit (Takara, Japan) on the StepOnePlus real-time fluorescence quantitative PCR system (Thermo Fisher, USA). The relative mRNA expression was calculated using the 2^–ΔΔCT^ method. The primer sequences are shown in Supplementary Material 3.

### Western blot analysis

For protein extraction from the tissue samples, the samples were first minced, and subsequently, RIPA lysis buffer and PMSF (Solarbio, China) were added. Samples were sonicated for 30 s, vortexed for 30 s, and incubated on ice for 5 min. This step was repeated thrice. The culture medium was discarded, and the cells were washed thrice with pre-cooled PBS on ice for protein extraction from the cell samples. RIPA lysis buffer and PMSF were added, vortexed for 30 s, and incubated on ice for 5 min. This step was repeated thrice. Tissue and cell samples were centrifuged at 14,000 rpm for 20 min, and the supernatant was collected as the protein sample. The protein concentration was determined using a BCA protein concentration detection kit (Solarbio, China). Proteins were separated using electrophoresis and transferred onto polyvinylidene fluoride membranes (Millipore). The membranes were blocked with a protein-free rapid blocking solution (Yadase, China) for 30 min, followed by overnight incubation with primary antibodies at 4 °C. The membranes were washed thrice with TBST and incubated with secondary antibodies for 1 h. After washing with TBST, protein expression was verified using ECL luminescent solution. The companies from which the antibodies were procured are listed in Supplementary Material 4.

### Lentivirus and plasmid construction

Commercially available lentiviruses for ZIC2 knockdown (sh-ZIC2), LYPLA2 overexpression (oe-LYPLA2), and LYPLA2 knockdown (sh-LYPLA2) were synthesized by Hanbio Biotechnology. After transfection into HN6 and SCC-9 cell lines, the knockdown and overexpression efficiencies were verified using quantitative reverse transcription polymerase chain reaction (RT-qPCR) and western blotting. Stable cell lines were selected using puromycin. The overexpression plasmid of ZIC2 (oe-ZIC2) was synthesized by Hanbio Biotechnology and transfected using Thermo Fisher Lipofectamine 3000 and P3000. RT-qPCR and western blotting were performed 24 h and 48 h after transfection to verify the overexpression efficiency, and relevant phenotypic experiments were conducted.

### Immunohistochemistry

The collected tissues were fixed in 4% paraformaldehyde (Solarbio, China), paraffin-embedded, and prepared into sections, which were baked and dewaxed with an alcohol gradient. Antigen retrieval was performed using a sodium citrate antigen retrieval solution (Solarbio, China). The sections were then blocked, incubated with primary antibodies overnight at 4 °C, and incubated at room temperature for 30 min. They were washed thrice with PBS for 5 min each, and subsequently incubated with secondary antibodies at room temperature for 30 min and washed thrice with PBS for 5 min each. Color development was carried out using DAB staining solution, and the reaction was terminated with tap water. Nuclei were stained with hematoxylin, and differentiation was performed using hydrochloric acid alcohol. The sections were washed with tap water to turn the nuclei sky blue. Dehydration, transparency, and drying were performed, and the sections were sealed with neutral gum. After placing a cover slip, the slides were air-dried, observed, and photographed under a microscope.

### CCK-8 assay

Stably transfected cells were seeded in 96-well plates at a density of 2000 cells per well. The optical density (OD) values were measured once daily. During detection, 10 μL of the CCK-8 reagent (Solarbio, China) was added to 90 μL of complete medium, and cells were incubated at 37 °C for 2 h. Absorbance was measured at 450 nm using a BioTek spectrophotometer.

### Transwell and wound healing assay

To detect cell migration, 60,000 stably transfected cells were seeded into the upper chamber of the Transwell. Serum-free cell culture medium was added to the upper chamber, and complete medium containing 20% FBS was added to the lower chamber. To detect cell invasion, Matrigel (Beyotime, China) was added to the serum-free medium in the upper chamber. Conditions in the lower chamber were unchanged. After 48 h, the cells were fixed with 4% paraformaldehyde for 30 min, washed with PBS, and stained with crystal violet for 20 min. Images were captured under a fluorescence microscope, and the number of migrated and invaded cells was counted.

The wound healing assay was also used to assess cell migration ability. Successfully constructed stable cell lines were seeded in 24-well plates at a density of 8 × 10^4^ cells per well. Once the cells have reached confluence, a scratch was made using a 10 μl pipette tip. The cells were then cultured in serum-free medium. After 24 h, images were captured, and cell migration was evaluated.

### Sphere formation assay

A dry medium was prepared using 50X B27 (Gibco), recombinant epidermal growth factor (EGF), basic fibroblast growth factor (bFGF) (Peorotech), and DMEM/F12 basal medium. Subsequently, 5000 cells per well were seeded into ultra-low attachment 24-well plates (LABSELECT, China), and 2 mL of dry medium was added to each well. After 14 d, the cells were observed, photographed, and counted.

### Drug sensitivity assay

Stock solutions (1 mM) of cisplatin and 5-fluorouracil (Topscience, Shanghai, China) were prepared. Working solutions were prepared by mixing the stock solutions with the culture medium at concentrations of 0.1, 1, 10, and 20 μM. In 96-well plates, 5000 cells were seeded per well. After 24 h, different drug concentrations were added. After 72 h, the medium was removed, and 90 μL of complete medium and 10 μL of CCK-8 solution were added to each well. OD was measured after 2 h.

### RNA-Seq analysis

Samples were collected after transfection, each containing 1 × 10^7^ cells. Total RNA quality was assessed, and the concentration and purity were measured using a Nanodrop 2000 spectrophotometer (Thermo Scientific). Total RNA ( ≥ 1 μg) was used. The NEBNext Ultra II RNA Library Prep Kit (Illumina) was used to enrich polyA-tailed mRNA using oligo (dT) magnetic beads. The mRNA was randomly fragmented using divalent cations. cDNA was synthesized using the fragmented mRNA as a template and random oligonucleotides as primers. Double-stranded cDNA was purified and ligated using sequencing adapters. Ampure XP beads were used to select cDNA fragments of lengths of approximately 400–500 bp. PCR amplification was performed, and the PCR products were purified using AMPure XP beads. The final libraries were obtained. Multiplex DNA libraries were normalized and mixed in equal volumes. The mixed libraries were quantified using serial dilution and sequenced on an Illumina sequencer in the PE150 mode.

### Targeted metabolomics analysis

Stable cell lines with the lentivirus were cultured in 10-cm dishes. After the cells covered the dish, they were digested and counted, ensuring that each sample in the detection group contained 1 × 10^7^ cells. The other cells to be tested were washed thrice with pre-cooled PBS, and 1 mL of pre-cooled PBS was added. The cells were scraped off with a cell scraper, flash-frozen in liquid nitrogen, and stored at –80 °C. After all samples were collected, they were thawed slowly at 4 °C. An appropriate amount of the sample was added to a pre-cooled methanol/acetonitrile/water solution (2:2:1) and vortexed. The mixture was subjected to low-temperature ultrasonication for 30 min, followed by a 10-min incubation at –20 °C. The samples were centrifuged at 14,000 × *g* for 20 min at 4 °C. The supernatant was vacuum-dried and subjected to mass spectrometry. Next, 100 μL of acetonitrile/water solution (acetonitrile: water = 1:1, v/v) was added to re-dissolve the sample. The mixture was vortexed and centrifuged at 14,000 × *g* for 15 min at 4 °C. The supernatant was used for injection. The samples were separated using an Agilent 1290 Infinity LC ultra-high-performance liquid chromatography system and a C18 chromatographic column, maintained at 4 °C in an automatic sampler, and analyzed randomly. QC samples were inserted into the sample queue to monitor and evaluate the stability of the system and the reliability of the experimental data. Mass spectrometry analysis was conducted, and peak extraction was performed using the raw data to obtain the ratio of the peak area of each substance to the peak area of the internal standard. The protein content was calculated using a standard curve.

### Tumors in nude mice

All nude mice used in this experiment were purchased from Beijing Sibeifu Biotechnology Co., Ltd, and the LYPLA2 inhibitor ML349 was obtained from MCE. Twenty-five male nude BALB/c mice (weighing 19–21 g and aged 4–5 weeks) were housed in the Laboratory Animal Center of Hebei Medical University under specific pathogen-free conditions. HN6 cell lines stably transfected with sh-NC, sh-ZIC2, or sh-LYPLA2 lentiviruses were collected. The mice were randomly divided into five groups (*n* = 5 per group) using a random number table: sh-NC, sh-ZIC2, sh-LYPLA2, NC-ML349 (3 mg/kg), and NC-ML349 (6 mg/kg). Each mouse was injected subcutaneously with 1 × 10^6^ cells resuspended in 1× PBS and mixed 1:1 with Matrigel. ML349 was added to the NC-ML349 (3 mg/kg) and NC-ML349 (6 mg/kg) groups. After tumor formation in nude mice, ML349 cells were administered at 3 and 6 mg/kg in the animals. The tumor volume did not exceed 2000 mm^3^. The tumor size was measured using a caliper, and the volume was calculated as *V* = 1/2×a×b^2^ (where *a* is the length and *b* is the width). The tumor was weighed using a balance.

### Detection of the tumorigenicity of cancer cell spheres

In the HN6 cell line, stable ZIC2 knockdown (sh-ZIC2) and control (sh-NC) cell lines were screened. After trypsin digestion, tumor cells were collected by centrifugation and cultured in stemness medium consisting of 50× B27, EGF, bFGF and DMEM/F12 basal medium in ultra-low attachment culture plates to form tumor spheres.

In vitro: After 7 days, tumor cell spheres from the control and knockdown groups were collected, re-plated in ultra-low attachment plates, and cultured in stemness medium. Cells were divided into three groups: sh-NC group, sh-ZIC2 group, and sh-ZIC2-GPC group. In the sh-ZIC2-GPC group, 10 μM GPC was added 24 h after re-plating. After 72 hours of re-plating, the tumor cell spheres from all three groups were filtered through a 70 µm filter and collected separately. Sphere counts were measured using a cell counter. For each group, three concentrations—2 × 10^2^, 2 × 10^3^, and 2 × 10^4^—were prepared, yielding nine subgroups in total.

In vivo: Subsequently, 36 nude mice were blindly assigned into 9 subgroups (4 mice in each subgroup) using a random number table. Each concentration of tumor spheres from the sh-NC, sh-ZIC2, and sh-ZIC2-GPC groups was injected into 4 mice. Tumor spheres mixed 1:1 with Matrigel were injected into the axilla of male BALB/c nude mice (weighing 19–21 g, aged 4–5 weeks old). Tumor formation was evaluated 6 weeks after injection.

### Statistical analysis

GraphPad Prism 9.0.0 and SPSS 26 were used for statistical analyses. Results were obtained from three independent experiments, data were expressed as mean ± standard deviation (SD). Student’s *t* test was used to analyze differences between two groups, and one-way analysis of variance (ANOVA) or two-way ANOVA was used for comparison among more than two groups. Statistical significance was set at *P* < 0.05.

## Results

### ZIC2 is highly expressed in OSCC and has poor prognosis

**Fig. 1:**
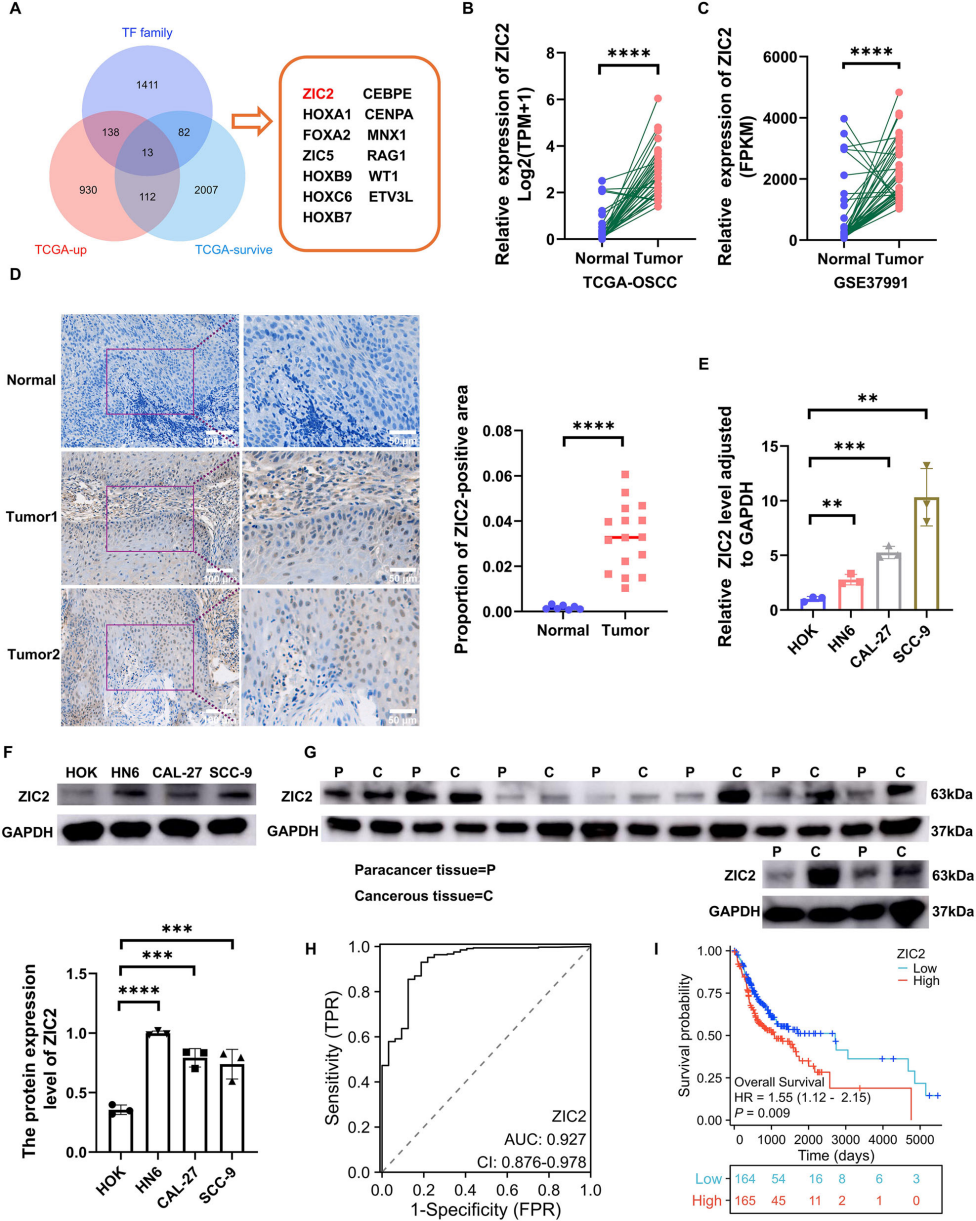
ZIC2 is highly expressed in OSCC and indicates a poor prognosis. **A** Venn diagram showing the intersection of upregulated genes in TCGA-OSCC, TF transcription factor families, and TCGA-OSCC prognosis-related genes. **B** Expression of ZIC2 in TCGA-OSCC, data were analyzed using Student’s *t* test, and the results were presented as mean ± SD. **C** Expression of ZIC2 in GSE37991, data were analyzed using Student’s *t* test, and the results were presented as mean ± SD. **D** IHC experiments for ZIC2 expression in OSCC and gingival tissues, data were analyzed using Student’s *t* test, and the results were presented as mean ± SD. **E** RT-qPCR to verify ZIC2 expression in HOK, CAL-27, HN6, and SCC-9 cell lines, data were analyzed using Student’s *t* test, and the results were presented as mean ± SD, *n* = 3. **F** western blot to verify ZIC2 expression in HOK, CAL-27, HN6, and SCC-9 cell lines, data were analyzed using one-way ANOVA, and the results were presented as mean ± SD, *n* = 3. **G** western blot experiments to verify ZIC2 expression in 9 OSCC and adjacent samples. **H** AUC value for ZIC2 expression. **I** Prognostic analysis of ZIC2 in OSCC samples in the TCGA database (ns no significant difference; *p < 0.05; **p < 0.01; ***p < 0.001; ****p < 0.0001).

TCGA was used to obtain OSCC samples, transcription factors database and TCGA-OSCC prognosis-related genes to identify transcription factors related to OSCC prognosis. A Venn diagram was used to obtain the intersection, yielding 13 OSCC prognosis-related transcription factors (Fig. 1A). Subsequently, by analyzing 32 OSCC and their paired tissue samples from TCGA, as well as 40 paired OSCC samples from the GEO dataset GSE37991, ZIC2 was found to be highly expressed in OSCC (Fig. 1B, C). Then, immunohistochemical analysis was performed on 15 OSCC and 7 gingival samples (Fig. 1D). RT-qPCR experiments (Fig. 1E) were conducted to detect the expression of ZIC2 in normal HOK and OSCC cell lines (HN6, CAL-27 and SCC-9), which also confirmed the high expression of ZIC2 in OSCC. Additionally, Western blot experiments were used to verify the expression of ZIC2 in cells and tissues (Fig. 1F, G). ROC curve analysis showed that the area under the curve (AUC) for ZIC2 expression was >0.9 (Fig. 1H), and high expression was significantly associated with poor prognosis in patients (Fig. 1I). Prognostic analysis of 12 differentially expressed genes is shown in Supplementary Figure 1. Our results suggest that ZIC2 is a potential diagnostic marker for OSCC.

### ZIC2 knockdown inhibits OSCC proliferation, migration, invasion, and sphere formation, while restoring chemotherapy sensitivity

**Fig. 2:**
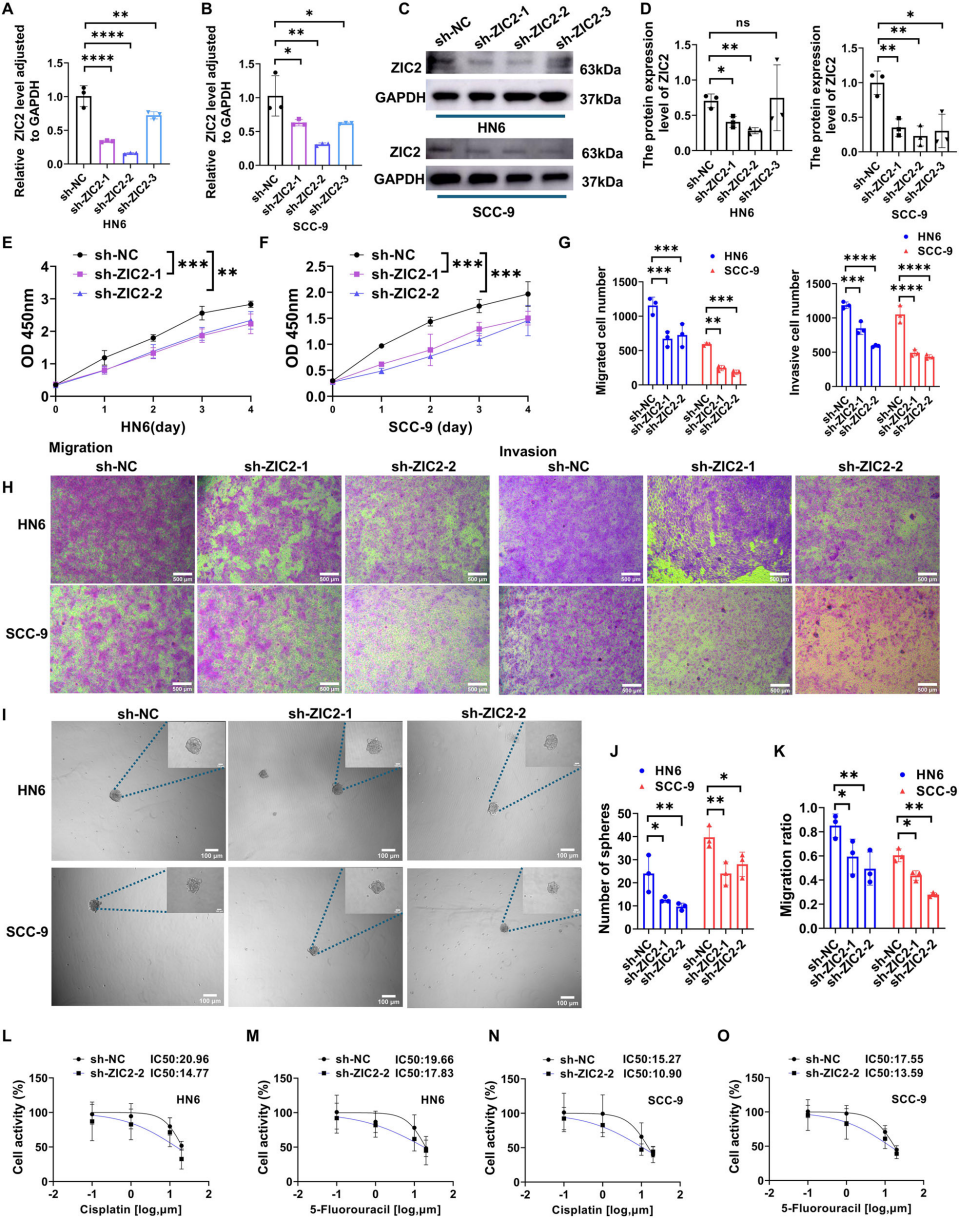
Knockdown of ZIC2 inhibits the progression of OSCC. **A**, **B** RT-qPCR experiments to verify the knockdown of ZIC2 at the gene level, data were analyzed using one-way ANOVA, and the results were presented as mean ± SD, *n* = 3. **C**, **D** Western blot experiments to verify the knockdown level of ZIC2 at the protein level, data were analyzed using Student’s *t* test, and the results were presented as mean ± SD, *n* = 3; **E**, **F** CCK-8 experiments after knocking down ZIC2, data were analyzed using two-way ANOVA, and the results were presented as mean ± SD, *n* = 3. **G**, **H** Transwell experiments after knocking down ZIC2, data were analyzed using two-way ANOVA, and the results were presented as mean ± SD, *n* = 3. **I**, **J** Sphere formation ability following ZIC2 knockdown, data were analyzed using two-way ANOVA, and the results were presented as mean ± SD, *n* = 3. **K** Statistical analysis for the wound healing assay after knocking down ZIC2, data were analyzed using two-way ANOVA and Student’s t-test, and the results were presented as mean ± SD, *n* = 3. **L**–**O** Detection of sensitivity to cisplatin and 5-fluorouracil in HN6 and SCC-9 cell lines after knocking down ZIC2 (ns, no significant difference; *p < 0.05; **p < 0.01; ***p < 0.001; ****p < 0.0001).

HN6 and SCC-9 cells were transfected with sh-ZIC2 lentivirus, and stable cell lines were established using puromycin to assess the role of ZIC2 expression in OSCC. RT-qPCR (Fig. 2A, B) and western blotting (Fig. 2C, D) were used to verify the effect of ZIC2 knockdown at the gene and protein levels. Subsequently, we selected the first two knockdown sequences for CCK-8 experiments and found that ZIC2 knockdown reduced the proliferative ability of OSCC cells (Fig. 2E, F). Transwell and wound healing assays revealed decreased migration and invasion abilities of OSCC cells following ZIC2 knockdown (Fig. 2G, H, K, and Supplementary Fig. 2). It has been reported that ZIC2 can regulate the stemness of ovarian tumor cells [18]. Therefore, we performed a microsphere formation assay and found that ZIC2 knockdown reduced the number of OSCC cell spheres (Fig. 2I, J). Cisplatin and 5-fluorouracil are chemotherapeutic drugs commonly used to treat OSCC [24]. After knocking down ZIC2, the IC50 values of cisplatin and 5-fluorouracil in cells decreased, indicating increased sensitivity to chemotherapy drugs (Fig. 2L–O).

### ZIC2 overexpression enhances OSCC proliferation, migration, invasion, and sphere formation, and reduces sensitivity to chemotherapy

**Fig. 3:**
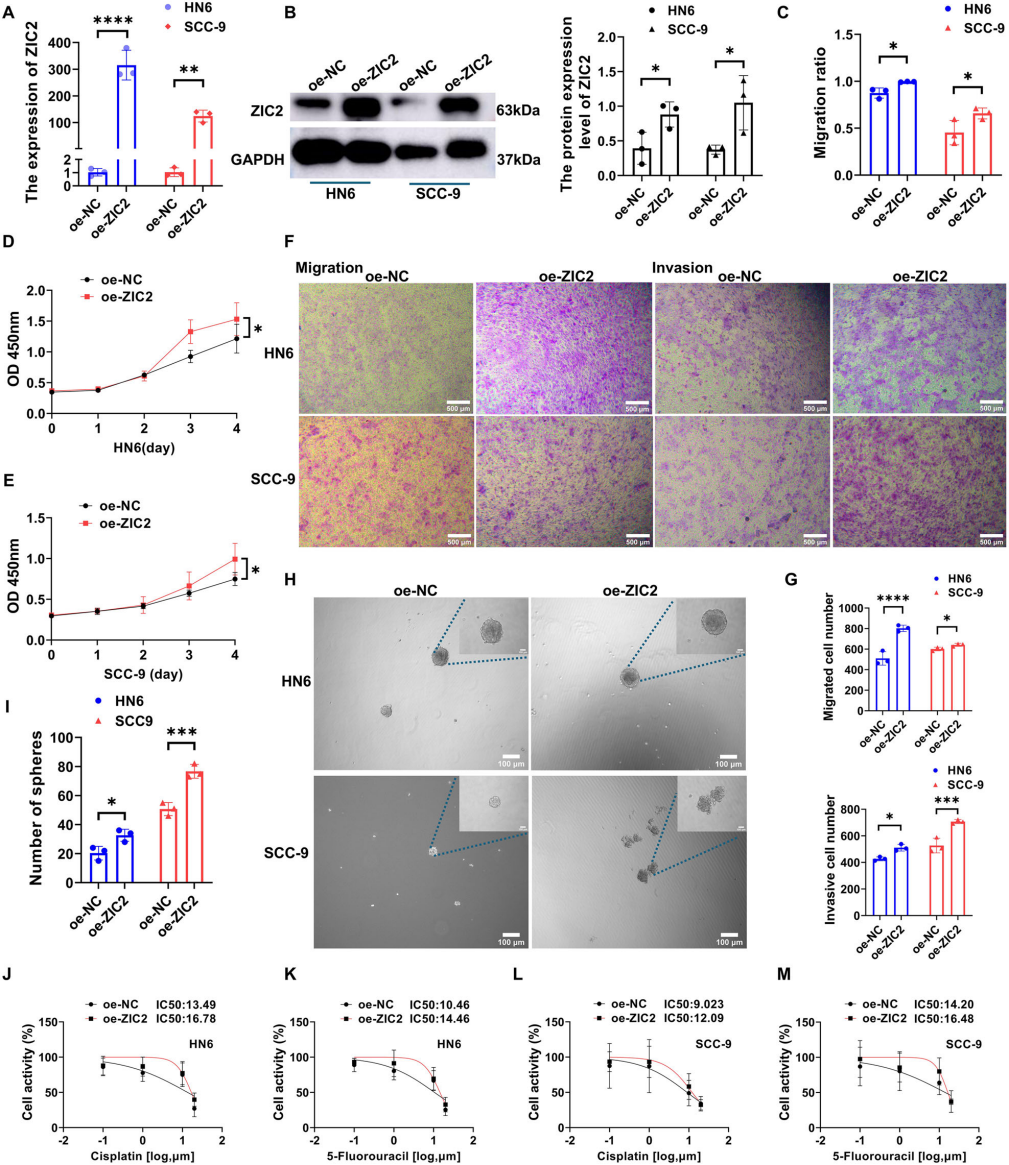
Overexpression of ZIC2 promotes the progression of OSCC. **A** RT-qPCR to verify the overexpression of ZIC2, data were analyzed using two-way ANOVA, and the results were presented as mean ± SD, *n* = 3. **B** Western blot to verify the overexpression of ZIC2, data were analyzed using two-way ANOVA and Student’s t-test, and the results were presented as mean ± SD, *n* = 3. **C** Statistical analysis of the wound healing assay after ZIC2 overexpression, data were analyzed using two-way ANOVA and Student’s t-test, and the results were presented as mean ± SD, *n* = 3. **D**, **E** CCK-8 assay following ZIC2 overexpression, data were analyzed using two-way ANOVA, and the results were presented as mean ± SD, *n* = 3. **F**, **G** Transwell assay following ZIC2 overexpression, data were analyzed using two-way ANOVA and Student’s *t* test, and the results were presented as mean ± SD, *n* = 3. **H** Detection of spheroid formation ability after overexpression of ZIC2; **I** Statistical analysis for spheroid formation after overexpression of ZIC2, data were analyzed using two-way ANOVA, and the results were presented as mean ± SD, *n* = 3. **J**–**M** Detection of drug sensitivity to cisplatin and 5-FU in HN6 and SCC-9 cell lines following ZIC2 overexpression (ns, no significant difference; *p < 0.05; **p < 0.01; ***p < 0.001; ***p < 0.0001).

We transfected HN6 and SCC-9 cell lines with oe-ZIC2 plasmids and verified the increased expression at gene and protein levels using RT-qPCR and western blotting, respectively (Fig. 3A, B). The CCK-8 assay showed that ZIC2 overexpression increased the proliferative ability of OSCC cells on the fourth day (Fig. 3D, E). The wound healing assay revealed that ZIC2 overexpression increased the migratory ability of OSCC cells (Fig. 3C, Supplementary Fig. 3). Transwell assays showed that ZIC2 overexpression increased the migration and invasion abilities of OSCC cells (Fig. 3F, G). Simultaneously, ZIC2 overexpression increased the sphere formation ability of OSCC (Fig. 3H, I) and increased the IC50 values of cisplatin and 5-fluorouracil (Fig. 3J–M). These results indicate the important role of ZIC2 in regulating the proliferation, migration, invasion, and sphere formation ability of OSCC cells, as well as drug sensitivity.

### ZIC2 regulates GPC metabolism in OSCC

**Fig. 4:**
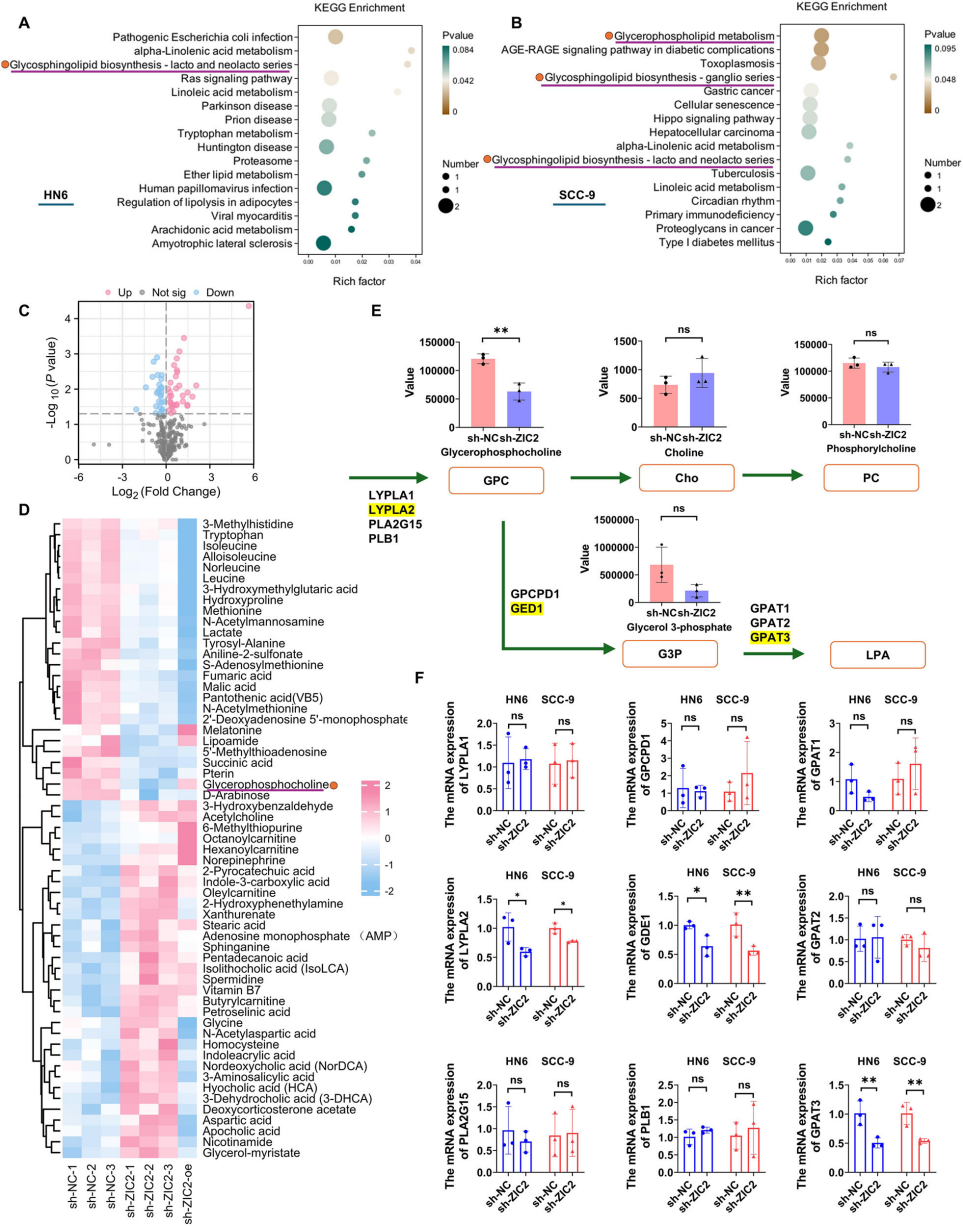
Knockdown of ZIC2 reduces the expression of glycerophosphocholine in OSCC. **A**, **B** KEGG enrichment analysis for differentially expressed genes in HN6 and SCC-9 cell lines. **C**, **D** Volcano plot and heatmap analysis of differential metabolites in targeted metabolomics. **E**, **F** Analysis of metabolite content in the GPC metabolic pathway and RT-qPCR detection of related rate-limiting enzymes, data were analyzed using two-way ANOVA and Student’s *t* test, and the results were presented as mean ± SD, *n* = 3 (ns, no significant difference; *p < 0.05; **p < 0.01; ***p < 0.001; ****p < 0.0001).

To further assess the role of ZIC2 in OSCC, we performed RNA-seq analysis for stable knockdown and control groups of HN6 and SCC-9 cells transfected with lentivirus. The screening criteria were |log2FC | ≥ 1.5 and *p*val. ≤ 0.05. We identified 39 differentially expressed genes in HN6 cells and 83 in SCC-9 cells (Supplementary Fig. 4A, B). KEGG analysis of downregulated differentially expressed genes revealed enriched glycosphingolipid and glycerophospholipid metabolism pathways in HN6 and SCC-9 cell lines (Fig. 4A, B), suggesting the role of lipid metabolism in OSCC progression. Next, the HN6 stable knockdown cell line was transfected with ZIC2 overexpression plasmids. After 48 h, samples were collected for ZIC2 knockdown, followed by overexpression. Subsequently, the stable ZIC2 knockdown cells and control cells were collected for targeted metabolomic analysis. GPC expression was downregulated after ZIC2 knockdown and upregulated after ZIC2 overexpression (Fig. 4C, D), indicating that GPC plays an important role in the progression of OSCC regulated by ZIC2. Next, we analyzed the GPC metabolic pathway and targeted metabolomics data. Choline and phosphatidylcholine expression did not change after ZIC2 knockdown, After knocking down the ZIC2 gene, although there was no statistical significance in the content of glycerol-3-phosphate (G3P) between the knockdown group and the control group, the actual detected values showed a downward trend. Therefore, we conducted RT-qPCR experiments on the rate-limiting enzymes in the metabolic pathway from GPC to glycerol-3-phosphate. LYPLA2, GED1, and GPAT3 were downregulated after ZIC2 knockdown, whereas LYPLA1, PLA2G15, PLB1, GPCPD1, GPAT1, and GPAT2 levels remained unchanged (Fig. 4E, F). These studies suggest that ZIC2 may regulate LYPLA2 and thereby affect GPC metabolism.

### ZIC2 affects the proliferation, migration, and invasion of OSCC cells by regulating LYPLA2

**Fig. 5:**
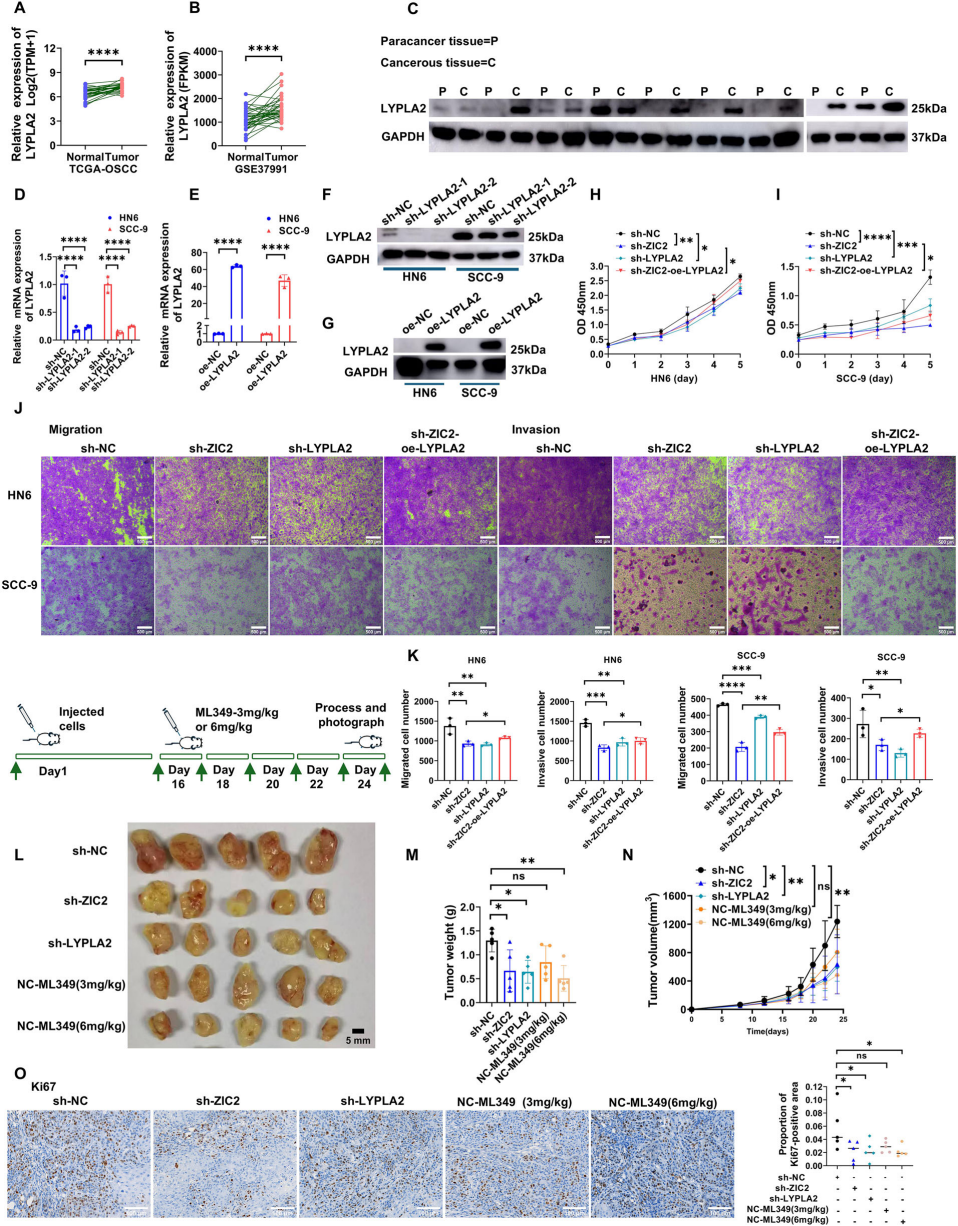
ZIC2 affects the progression of OSCC by mediating LYPLA2. **A** Expression of LYPLA2 in TCGA-OSCC. **B** Expression of LYPLA2 in GSE37991. **C** Western blot to verify LYPLA2 expression in 9 OSCC and adjacent samples. **D**, **E** RT-qPCR to verify knockdown and overexpression of LYPLA2, data were analyzed using two-way ANOVA, and the results were presented as mean ± SD, *n* = 3. **F**, **G** Western blot to verify knockdown and overexpression of LYPLA2. **H**, **I** CCK-8 experiments in HN6 and SCC-9 cell lines, data were analyzed using one-way ANOVA and Student’s *t* test, and the results are presented as mean ± SD, *n* = 3. **J**, **K** Transwell experiments in HN6 and SCC-9 cell lines, data were analyzed using one-way ANOVA and Student’s *t* test, the results were presented as mean ± SD, *n* = 3. **L**–**N** Images of transplanted tumors in nude mice and statistical analysis of tumor weight and volume in each group of mice were conducted, data were analyzed using one-way ANOVA, and the results were presented as mean ± SD, *n* = 3. **O** Detection of the Ki67 index in each group of mice, data were analyzed using one-way ANOVA, and the results were presented as mean ± SD, *n* = 5 (ns, no significant difference; *p < 0.05; **p < 0.01; ***p < 0.001; ****p < 0.0001).

We explored the role of LYPLA2, a key enzyme in GPC synthesis, in regulating OSCC progression by ZIC2. We first detected the expression of LYPLA2 in OSCC samples from TCGA and GSE37991 datasets and found high expression of LYPLA2 in OSCC (Fig. 5A, B). This was verified using western blot analysis of 9 pairs of OSCC and adjacent samples (Fig. 5C). Next, we constructed lentiviruses that stably knocked down LYPLA2 and overexpressed LYPLA2 and verified their knockdown and overexpression efficiency using RT-qPCR (Fig. 5D, E) and western blotting (Fig. 5F, G). We selected the second sequence to knockdown LYPLA2 to establish a cell line with stable LYPLA2 knockdown and added the overexpressing LYPLA2 lentivirus to the stable knockdown ZIC2 cell line to construct the sh-ZIC2-oe-LYPLA2 stable cell line. CCK-8 assay showed that knocking down LYPLA2 reduced the proliferative ability of OSCC cells, while LYPLA2 overexpression rescued the effect of ZIC2 knockdown on proliferation (Fig. 5H, I). Wound healing assay and Transwell assays showed that knocking down LYPLA2 reduced the migration and invasion abilities of OSCC cells, while LYPLA2 overexpression rescued the effect of knocking down ZIC2 on migration and invasion (Fig. 5J, K, Supplementary Fig. 5). Subsequently, we established an animal model using BALB/c nude mice. ML349 as a LYPLA2 inhibitor. ML349 was added to the NC-ML349 (3 mg/kg) and NC-ML349 (6 mg/kg) groups 16 days after tumor cell injection. Knocking down ZIC2 and LYPLA2 inhibited the rate and weight of OSCC tumors. The inhibitory effect on tumors did not differ when the inhibitor was added at 3 mg/kg; however, a statistically significant difference was observed at 6 mg/kg. Detection of the Ki67 index in tumor tissue samples verified this conclusion (Fig. 5L–O). Thus, we infer that LYPLA2 is important in regulating OSCC progression through ZIC2.

### GPC can increase the stemness of OSCC

**Fig. 6:**
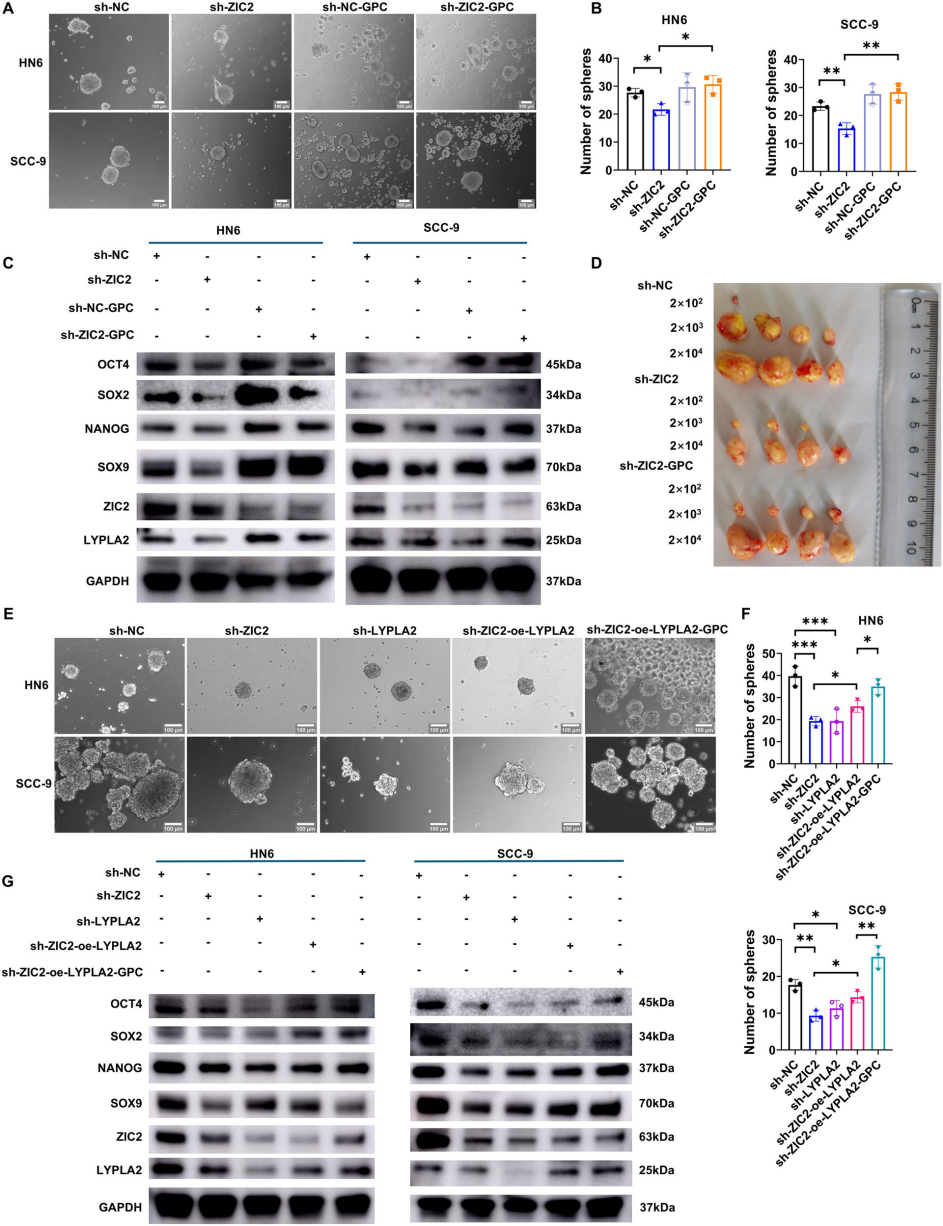
ZIC2 regulates LYPLA2 to influence glycerophosphocholine metabolism and thereby control the stemness of OSCC. **A**, **B** Spheroid formation ability in HN6 and SCC-9 cell lines, data were analyzed using Student’s *t* test, and the results were presented as mean ± SD, *n* = 3. **C** Western blot detection of tumor stemness markers in HN6 and SCC-9 cell lines. **D** Cancer cell sphere tumorigenicity. **E**–**G** Spheroid formation ability and western blot detection of tumor stemness markers in HN6 and SCC-9 cell lines after GPC addition, data were analyzed using one-way ANOVA and Student’s *t* test, and the results were presented as mean ± SD, *n* = 3 (ns, no significant difference; *p < 0.05; **p < 0.01; ***p < 0.001; ****p < 0.0001).

To explore the role of GPC in the ZIC2-regulated progression of OSCC, we analyzed the lipid content and tumor cell stemness-related GSE72118 dataset. Gene set enrichment analysis (GSEA) showed that the related genes were enriched in glycosphingolipid metabolism and cholesterol and lipid homeostasis pathways (Supplementary Fig. 6A, B). Therefore, we hypothesized that GPC might be related to tumor stemness. Next, commercial GPC was used to detect cell viability in HN6 and SCC-9 lines with graded concentrations, and selected 10 μM as the working concentration (Supplementary Fig. 6C, D). The addition of GPC increased the number of cancer cell spheres, as evidenced by the microsphere formation assay (Fig. 6A, B). Tumor stemness markers, including OCT4, SOX2, Nanog, and SOX9, were downregulated following ZIC2 knockdown, and GPC addition restored their levels (Fig. 6C). We conducted animal experiments and found that the cancer cell spheres in the group with ZIC2 knockdown and GPC addition had a higher tumor formation rate and size compared to the group with only ZIC2 knockdown (Fig. 6D, Supplementary Fig. 6E). As the rate-limiting enzyme of GPC, LYPLA2 may be involved in ZIC2-mediated regulation of GPC and tumor cell stemness. The microsphere formation assay showed that the number of cancer cell spheres decreased after LYPLA2 knockdown (Fig. 6E, F). Overexpression of LYPLA2 after ZIC2 knockdown showed an increase in the number of cancer cell spheres, and GPC addition increased the number of cancer cell spheres compared to the group with no addition (Fig. 6E, F). Next, we detected the expression of tumor stem cell-related markers and found reduced expression of OCT4 and Nanog in HN6 and SCC-9 cell lines after LYPLA2 knockdown; the expression of SOX2 and SOX9 remained unchanged in HN6 cells but was downregulated in SCC-9 cells. After knocking down ZIC2 and overexpressing LYPLA2, SOX9 expression was increased; Nanog expression was elevated in the SCC-9 cell line but not obvious in the HN6 cell line; OCT4 expression was slightly upregulated in both cell lines; however, no effect on SOX2 expression was observed. Similarly, after knocking down ZIC2 and LYPLA2 overexpression, adding GPC, the expression of OCT4 and Nanog proteins increased, while that of SOX2 and SOX9 proteins was not significantly affected (Fig. 6G). Thus, ZIC2 may regulate GPC synthesis through LYPLA2, further increasing the expression of OCT4 and Nanog and maintaining the self-renewal ability of OSCC CSCs and affecting the progression of OSCC.

## Discussion

In this study, we identified an OSCC stemness-related transcription factor, ZIC2, and found that high ZIC2 expression was associated with poor prognosis in patients. We demonstrated that ZIC2 may affect the progression of OSCC by regulating the rate-limiting enzyme of GPC, LYPLA2. The addition of exogenous GPC increased the stemness of OSCC cells. Overall, our findings suggest that ZIC2 can regulate tumor cell stemness through the LYPLA2–GPC axis in OSCC, highlighting its potential as a therapeutic target.

CSCs are a subset of tumor cells involved in tumor growth, epithelial–mesenchymal transition (EMT), tumor metastasis, and tumor drug resistance [25]. Lipids are the basic components of cell membranes and play crucial roles in cell recognition, signal transduction, and energy supply [26]. Alterations in lipid metabolism can affect the self-renewal, differentiation, invasion, metastasis, and drug sensitivity of CSCs [27]. Phosphatidylcholine is hydrolyzed to generate free fatty acids and GPC. As a type of lipid, fatty acids serve as an energy source for CSCs. An increase in the expression of genes related to fatty acid oxidation has been observed in CSCs, which maintains the self-renewal of CSCs by mediating lipid and membrane synthesis, regulating NADPH production to quench reactive oxygen species, and promoting chemoresistance [28]. GPC metabolism, an important component of lipid metabolism, has been implicated in breast [29] and ovarian cancers [30]; however, its association with CSC characteristics has not been previously identified. In this study, bioinformatics and experimental validation were used to identify the transcription factor ZIC2, found to be associated with the stemness of OSCC, and providing a new perspective on CSCs.

The role of ZIC2 in CSCs has been reported. Zhu et al. reported high expression of ZIC2 in liver CSCs, playing a significant role in maintaining self-renewal. ZIC2 correlates positively with the clinicopathological stage of patients with HCC and can be used to diagnose and predict prognosis [19]. ZIC2 regulates the stemness of lung adenocarcinoma cells and affects their sensitivity to chemotherapeutic drugs, such as cisplatin and paclitaxel [31]. In this study, knocking down ZIC2 reduced the proliferation, migration, invasion, and tumor sphere formation abilities of OSCC cells, and lowered the IC50 values of cisplatin and 5-fluorouracil, thereby increasing drug sensitivity. This suggests that ZIC2 functions as a tumor stemness-related gene in OSCC. We performed RNA-seq and targeted metabolomic sequencing on ZIC2 knockdown cell lines and found decreased GPC content in tumor cells, suggesting that ZIC2 may influence OSCC progression by regulating lipid metabolism.

GPC, an important metabolic intermediate of the cholinergic system, participates in phospholipid metabolism and the synthesis of the acetylcholine neurotransmitter [32]. Glycerophosphodiesterase EDI3 (GPCPD1) hydrolyzes GPC into choline and 3-phosphoglycerol. Keller et al. reported the highest level of GPCPD1 expression in estrogen receptor (ESR1)-negative HER2-positive (ER^–^HER2^+^) human breast tumors. Silencing GPCPD1 reduces the viability of ER^–^HER2^+^ cells. In ER^–^HER2^+^ cells resistant to HER2-targeted therapy, silencing or pharmacological inhibition of GPCPD1 expression using dipyridamole can reduce cell viability and tumor growth [33]. GPC metabolism is a subtype of lipid metabolism. Wang et al. reported that ablation of Arf1 in mice disrupted lipid metabolism, leading to the accumulation of lipid droplets and metabolic stress that selectively eliminated CSC-enriched cells. The dying CSCs released damage-associated molecular patterns (DAMP), which activate dendritic cells (DCS), further acting on T cells to enhance anti-tumor immune function. Additionally, dying CSCs can also be transformed into therapeutic vaccines that act on immune cells and induce long-lasting therapeutic effects [34]. *Porphyromonas gingivalis* is a resident bacterial community in the oral cavity. In 2025, Zang et al. found that *P. gingivalis* upregulated the expression of stearoyl-coa desatase 1 (SCD1), a key enzyme in lipid metabolism, through the NOD1/KLF5 axis, which increased lipid synthesis in OSCC cells and ultimately promoted the acquisition of stemness [35]. These findings suggest that lipid metabolism plays an important role in the regulation of tumor stemness.

LYPLA2 is crucial in the stability of lipid metabolism [36]. We found that LYPLA2 played a significant role in GPC synthesis by analyzing the glycerophospholipid metabolism pathway in the KEGG database. In this study, by analyzing the metabolite content and rate-limiting enzymes in the GPC metabolic pathway, knocking down ZIC2 resulted in a decrease in the GPC content; glycerol-3-phosphate was also affected, and its level decreased. The levels of rate-limiting enzymes, such as LYPLA2, GED1, and GPAT3, also decreased following ZIC2 knockdown. These results suggest that ZIC2 affects OSCC progression through the LYPLA2/GPC/GEDI pathway. ZIC2 is associated with the stemness of various tumor cells, and our GSEA of related genes in the GSE72118 database showed the association of lipid metabolism with tumor stemness. The addition of GPC increased tumor cell stemness; LYPLA2, a key enzyme in the GPC synthesis pathway, was found to be involved in this mechanism. Ultimately, ZIC2 regulates LYPLA2 and acts on GPC, mediating changes in the expression of the tumor stemness markers Nanog and OCT4. This finding suggests that ZIC2 regulates tumor stemness markers in OSCC and acts as a potential therapeutic target for tumors.

In this study, we identified ZIC2 as an OSCC stemness-related gene and elucidated the mechanism by which it mediated changes in GPCs through LYPLA2, further enhancing the stemness of tumor cells. ZIC2 is a promising prognostic and diagnostic marker and may be a potential therapeutic target for OSCC. Although in vitro and in vivo experiments were conducted in this study, the mechanism by which CSCs, as a small but highly tumorigenic cell subpopulation of tumor cells, promote the rapid progression of OSCC has not been thoroughly investigated, which merits further exploration. Future research should focus on these aspects.

In summary, through bioinformatics and in vitro and in vivo experiments, ZIC2 was identified as a prognostically relevant gene in OSCC. ZIC2 knockdown reduced the expression of LYPLA2, thereby decreasing the expression of GPC metabolites in cells. GPC addition rescued the stemness of tumor cells after ZIC2 knockdown, increased the number of OSCC cancer cell spheres, and elevated the expression of the tumor stemness markers Nanog and OCT4. Our findings suggest that ZIC2 is a stemness-related gene in OSCC and a candidate target for treating OSCC.

## Supplementary information

Supplementary material

Supplementary Table 1

Supplementary Table 2

Supplementary Table 3

## Data Availability

The RNA-seq data have been deposited in the GEO database (GSE304366). The results of targeted metabolomics are provided in the supplementary materials 5, and detailed information can be obtained by contacting the corresponding author.
