# Supplementary material for: ZIC2 affects oral squamous cell carcinoma stemness by regulating glycerophosphocholine metabolism via LYPLA2
Source: Cell Death Dis. 2026 Mar 19;17(1):486. doi: 10.1038/s41419-026-08483-w (PMC13187452; doi:10.1038/s41419-026-08483-w)

Supplementary Material:

Fig1:

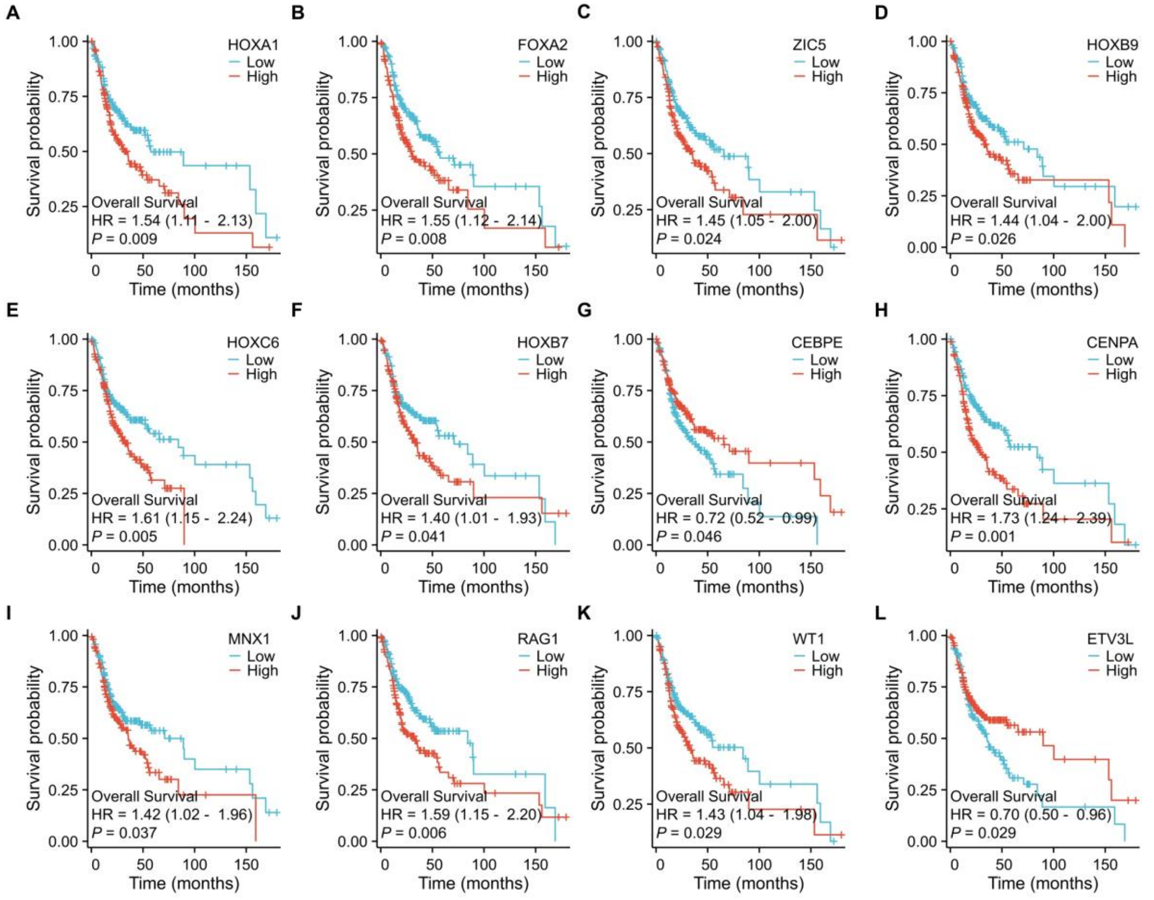

Supplementary Fig. 1. Prognostic analysis plots for the remaining 12 prognosis-related transcription factors.

Supplementary Material:

Fig2:

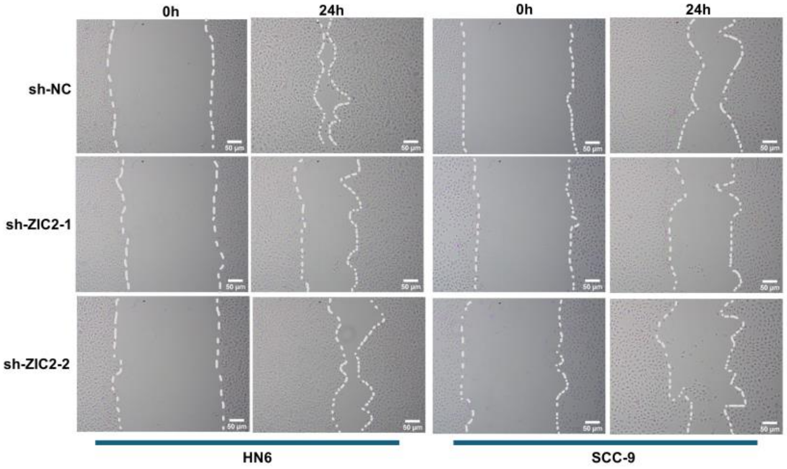

Supplementary Fig. 2. Wound healing assay in HN6 and SCC-9 cell lines following ZIC2 knockdown.

Supplementary Material:

Fig3:

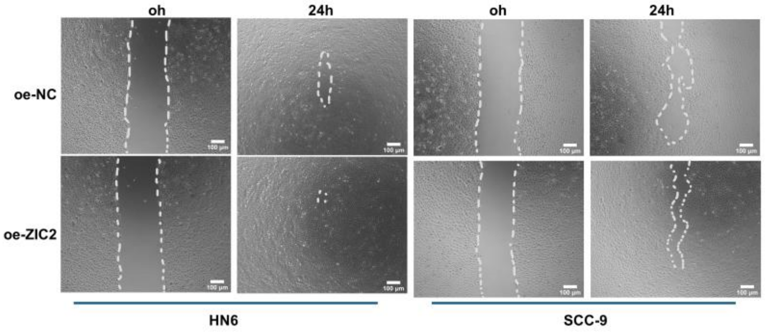

Supplementary Fig. 3. Wound healing assays in HN6 and SCC-9 cell lines after overexpressing ZIC2.

Supplementary Material:

Fig4:

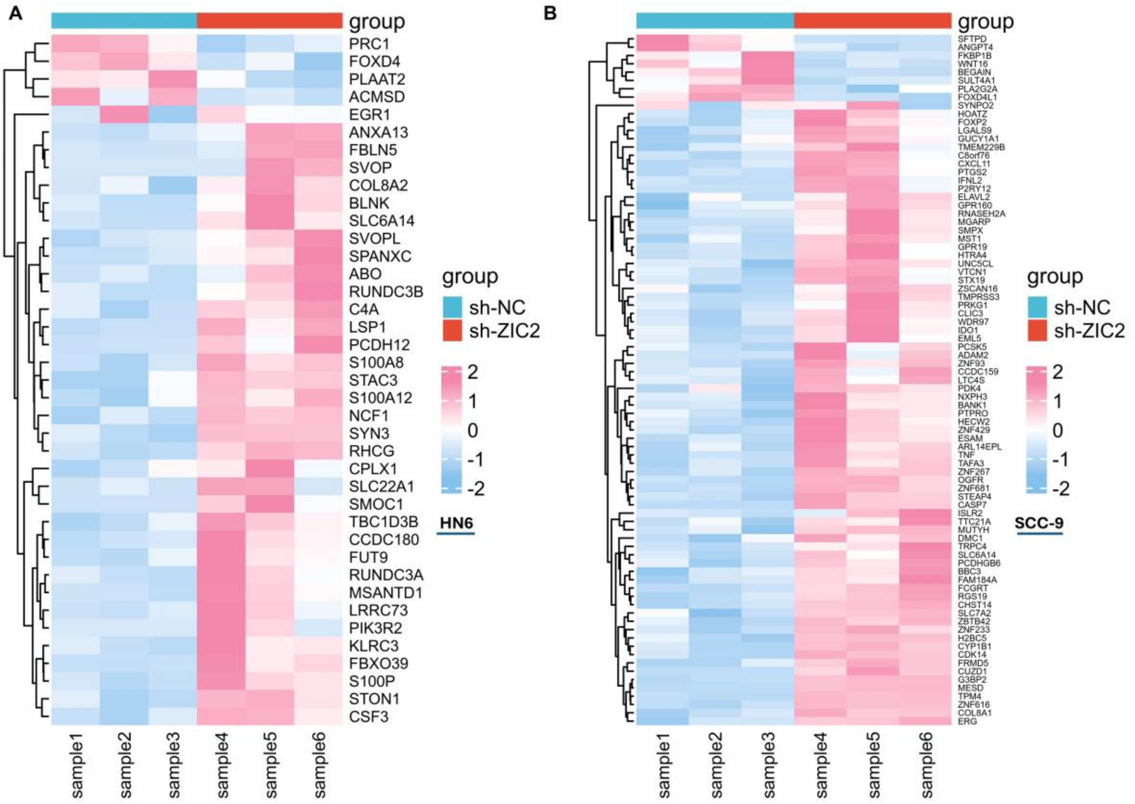

Supplementary Fig. 4: A–B. Heatmap showing differentially expressed genes in HN6 and SCC-9 cell lines.

Supplementary Material:

Fig5:

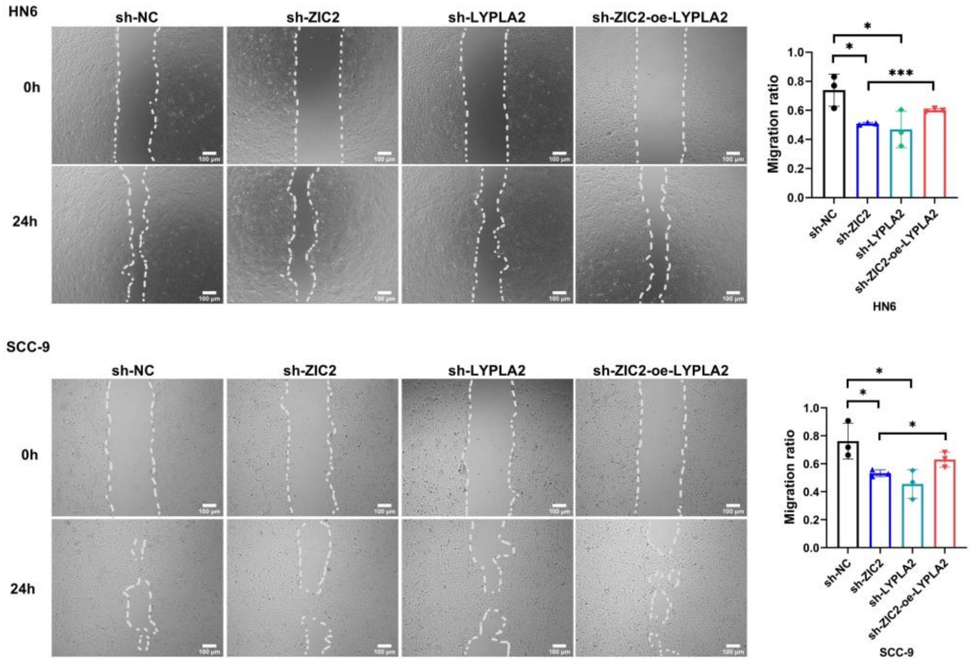

Supplementary Fig. 5. Wound healing assays and statistical analysis of rescue experiments in HN6 and SCC-9 cell lines; data were analyzed using one-way ANOVA, and the results are presented as mean ± SD, n = 3.

Supplementary Material:

Fig6:

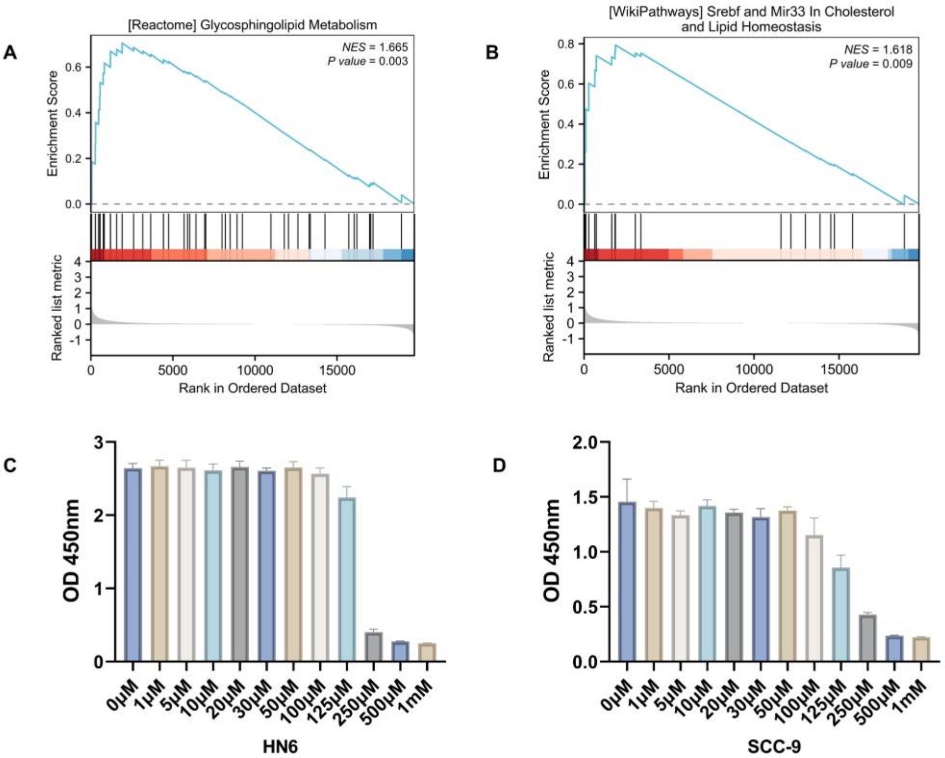

E

Number of tumors formed by cancer cell spheres/Total number of nude mice				
	Number of injected cell spheres	2 Weeks	4 Weeks	6 Weeks
sh-NC	2×10^2	0/4	0/4	1/4
	2×10^3	0/4	4/4	4/4
	2×10^4	1/4	4/4	4/4
sh-ZIC2	2×10^2	0/4	0/4	0/4
	2×10^3	0/4	2/4	3/4
	2×10^4	1/4	4/4	4/4
sh-ZIC2-GPC	2×10^2	0/4	0/4	0/4
	2×10^3	0/4	3/4	4/4
	2×10^4	1/4	4/4	4/4

Supplementary Fig. 6. A–B: GSEA of genes in the GEO dataset GSE72118; C–D: OD detection for glycerophosphocholine at different concentrations in HN6 and SCC-9 cell lines: E: Statistical analysis of the number and time of tumor formation in tumor cell spheres.

Supplementary material 1

Fifteen OSCC tissues

tumor	age	gender	Sampling site	diagnosis
1	83	female	gingiva	Squamous cell carcinoma of the lower gingiva
2	75	male	gingiva	Squamous cell carcinoma of gingiva
3	72	male	cheek	Squamous cell carcinoma of cheek
4	73	female	tongue	Squamous cell carcinoma of tongue
5	50	male	tongue	Squamous cell carcinoma of tongue
6	82	male	tongue	Squamous cell carcinoma of tongue
7	72	male	cheek	Squamous cell carcinoma of cheek
8	76	female	gingiva	Squamous cell carcinoma of gingiva
9	75	male	cheek	Squamous cell carcinoma of cheek
10	69	female	cheek	Squamous cell carcinoma of cheek
11	76	female	cheek	Squamous cell carcinoma of cheek
12	67	male	gingiva	Squamous cell carcinoma of gingiva
13	62	female	cheek	Squamous cell carcinoma of cheek
14	74	female	tongue	Squamous cell carcinoma of tongue
15	68	male	tongue	Squamous cell carcinoma of tongue

seven gingival tissues

normal	age	gender	Sampling site
1	19	male	gingiva
2	17	male	gingiva
3	25	female	gingiva
4	18	male	gingiva
5	22	female	gingiva
6	26	male	gingiva
7	28	male	gingiva

Supplementary material 2

Clinical information of 9 pairs of cancer patients and their adjacent non-cancerous counterparts

tumor	age	gender	Sampling site	diagnosis
1	39	female	gingiva	Carcinoma of the right lower gingiva
2	70	male	gingiva	Carcinoma of the left gingiva
3	55	female	Gingiva	Carcinoma of the maxillary gingiva
4	72	male	Cheek	Squamous cell carcinoma of cheek
5	72	male	gingiva	Carcinoma of the right lower gingiva
6	62	male	tongue	Squamous cell carcinoma of the left tongue
7	70	female	cheek	Squamous cell carcinoma of the left cheek
8	69	male	Floor of mouth	Squamous cell carcinoma of the left floor of the mouth
9	78	female	cheek	Squamous cell carcinoma of the left cheek

Supplementary material 3

ZIC2	FORWARD	CGAGCAACTGAGCAATCCCAAG
	REVERSE	GACACTCCTCCCAGAAGCAGAC
GPAT1	FORWARD	GATGCTGCTGATGAAGGTAGAGAC
	REVERSE	TGGACATAATGGCACAGGACTTG
GPAT2	FORWARD	TAGTGACAGTGATGACTTCGGAGAG
	REVERSE	CTGAGCAGGCGGCAGAGG
GPAT3	FORWARD	AAGCCATTGTAGAAGATGAAGTGACC
	REVERSE	ACCATAGTGAGCCGCAGACTG
GPCPD1	FORWARD	ACTCATCTACAACCACGATCAATAACC
	REVERSE	TGTCAGCCATCCAGAATCCAGAG
LYPLA1	FORWARD	GGTCCTATCGGTGGTGCTAATAGAG
	REVERSE	AAGGTCACATTGGCTGGATTCAC
LYPLA2	FORWARD	ATGAAGATGGTGATGCCCTCCTG
	REVERSE	CCTTGATGTTCTCTGCTGCCTTC
PLA2G15	FORWARD	TGTTCCTCTTGCTGCTGCTAATG
	REVERSE	GCACCACTGTCGGCTTGTC
PLB1	FORWARD	GGAGAAGCAAGACTGGACTGAAAG
	REVERSE	CACAGGCATTGGAACAGAAGGAC
GAPDH	FORWARD	CAGGAGGCATTGCTGATGAT
	REVERSE	GAAGGCTGGGGCTCATTT
GDEI	FORWARD	GCAGCAAACCACAGACTCAGG
	REVERSE	AGAGCCTCAGTAGCCTTGTGTG

Supplementary material 4

antigen	vendor	catalog number
ZIC2	ABCAM	ab150404
LYPLA2	ABclonal	A15792
SOX2	Abways	CY5268
SOX9	Abways	CY5400
Oct4	Abways	CY5781
Nanog	Abways	CY5611
GAPDH	Abways	AB0036

Supplementary material 5

Name	sh-NC-1	sh-NC-2	sh-NC-3	sh-ZIC2-1	sh-ZIC2-2	sh-ZIC2-3	sh-ZIC2-oe
Succinic acid	191820.9457	101911.8023	96558.88771	50147.66275	22826.95392	21031.65866	
Pterin	128.0193282	96.62965515	106.2691269	56.84959647	48.4277466	20.62216731	92.34784145
Glycerophosphocholine	122240.6647	128147.5158	111162.3139	76944.13615	47170.35419	64968.57823	179489.1045
Fumaric acid	474407.6997	414133.1009	427052.4567	269278.0927	251899.4504	207276.0096	256351.7053
Lipoamide	77.73018042	71.87790067	107.1910392	46.53354766	49.04380734	61.09149219	67.92961044
N-Acetylmethionine	25709.89609	19770.17173	19628.09676	15990.94461	10749.37644	13675.67015	16122.31211
Tyrosyl-Alanine	23247.85426	27028.90661	27706.72028	17388.94749	13498.21567	19183.19141	25772.92206
Aniline-2-sulfonate	386.5824801	418.1063621	368.2523503	240.4586597	251.0769516	270.0676978	302.7366883
N-Acetylmannosamine	889905.5568	738710.8503	818527.5439	557451.1292	524538.6814	558625.0309	699726.7601
Malic acid	1142837.077	929710.637	943103.0274	718964.1331	698974.7309	630345.5918	740308.3806
2'-Deoxyadenosine 5'-monophosphate(dAMP)	1807.226033	1485.477089	1557.217781	1158.8552	1073.316251	1198.509162	1745.117988
Pantothenic acid(VB5)	743780.563	574769.2026	617556.7492	466192.7053	467008.3425	449594.8461	1789356.326
5'-Methylthioadenosine	4232.551137	4688.848111	5054.075726	3173.83279	3491.681705	3442.417754	3105.678084
Lactate	4243074.893	3250745.993	3956511.646	2810217.803	2766506.955	2978130.639	1104558.496
Hydroxyproline	27957.5196	24927.7304	25546.7461	20894.21567	18098.84128	21850.6574	19288.75266
Melatonine	0.491364368	0.546396057	0.48378503	0.371400417	0.432138373	0.380987925	
S-Adenosylmethionine	57326.95978	57558.17704	45648.68557	42565.83133	39407.34103	43382.01968	51829.63128
Methionine	1761670.867	1603026.671	1694208.172	1361334.842	1264528.198	1404541.656	1471481.19
Norleucine	4151681.919	3602022.126	4048944.689	3069746.618	3101898.206	3278882.185	2908792.404
Tryptophan	2603026.616	2469611.286	2685989.787	1947429.727	2240955.69	2037760.199	2297040.533
Isoleucine	1932053.536	1737913.796	1958888.028	1474512.689	1463032.26	1581055.587	1397111.471
Leucine	2716218.769	2465977.986	2691063.453	2097589.834	2107439.182	2178012.671	1969529.569
Alloisoleucine	1937242.599	1675052.905	1955748.738	1473035.575	1468209.936	1582215.46	1357875.847
3-Hydroxymethylglutaric acid	19488.58802	16538.71766	17596.03219	15387.86955	13420.4153	15271.59545	32900.43469
D-Arabinose	3317318.784	3366811.061	3383682.862	3023769.917	2448030.435	2935247.625	2402455.447
3-Methylhistidine	1625.244617	1583.62121	1668.374653	1433.647607	1486.223344	1537.848938	
2-Pyrocatechuic acid	3837.571045	3662.030201	3612.587586	4160.313213	3997.15885	4145.332797	4139.969264
Homocysteine	5587.962149	6124.483021	5722.704107	6596.135027	6396.241506	7296.290836	7513.796822
Adenosine monophosphate （AMP）	16617.34065	17692.98204	15821.3364	20150.86997	20621.54077	18746.49135	20286.1694
Hyocholic acid (HCA)	43962.47879	41977.46508	38353.04542	51827.04065	48321.12565	49374.0737	53538.38857
Glycine	1186711.944	1139976.595	1016486.793	1396043.91	1388152.213	1268627.44	642864.6648
Aspartic acid	213442.1258	209577.3111	213682.8214	232313.715	273217.9146	268242.0731	518369.293
6-Methylthiopurine	2.168879986	2.162204693	2.385722244	2.649375992	2.744552345	2.884354481	
3-Hydroxybenzaldehyde	343.9333206	384.4959552	414.5228618	472.5714406	515.3399738	466.4616502	
Deoxycorticosterone acetate	23.60895165	19.74842367	24.9591177	30.20612496	25.90585971	31.09531756	
Indoleacrylic acid	20.39973498	23.19875629	17.64706137	25.71157236	25.32901314	28.4515874	
N-Acetylaspartic acid	29728.31421	25993.71048	25369.04052	38537.36766	31715.16351	35306.64101	55367.6624
Acetylcholine	8.176922686	9.986000194	11.06111143	11.98487489	13.42793589	14.27471283	43.64116401
Nordeoxycholic acid (NorDCA)	4509.649129	3842.608983	2976.03184	5807.28027	4761.60244	5451.459679	4586.861711

Supplementary material 5

3-Aminosalicylic acid	572.031668	541.7738863	375.7391731	747.6194048	677.133932	713.0508677	
3-Dehydrocholic acid (3-DHCA)	3163.725441	2878.560836	2674.855228	4386.621592	4333.570365	3993.157218	3931.233614
Apocholic acid	3057.622476	2389.171512	2556.727137	3917.483768	4533.029951	4234.980652	2029.216755
Nicotinamide	22234.98949	25811.84469	31380.00591	46171.84174	42864.27894	38579.34886	42972.27431
Hexanoylcarnitine	519.3555585	380.6775874	491.0376911	602.8831785	838.8278115	822.5059426	2560.652007
Vitamin B7	14.66877907	11.23438202	13.89516887	21.32280606	22.2380391	22.52158277	
Octanoylcarnitine	26.85971749	33.5647477	49.60956095	62.0921501	64.7549264	56.62838833	113.6815848
Oleylcarnitine	5956.12566	6995.284336	5840.920754	9567.553864	10412.77989	11410.84089	22229.95498
Butyrylcarnitine	5311.272493	3758.197791	4548.871674	7336.258549	8069.227523	7594.044564	9998.809406
Glycerol-myristate	2034.350149	3116.051607	3300.950251	4991.858945	5842.451191	4912.396155	10005.34959
2-Hydroxyphenethylamine	29016.88991	21259.44663	26281.08571	46194.18739	48005.58353	49875.38414	43331.95528
Pentadecanoic acid	48349.95998	49715.48082	47779.19453	85139.93226	134070.7769	101896.6763	71410.45738
Isolithocholic acid (IsoLCA)	20653.87168	10432.12919	18029.05185	29553.03309	45037.35124	34353.96239	42334.31916
Xanthurenate	636.1107988	461.9898269	627.279085	1267.844708	1389.004739	1375.211386	1087.087178
Stearic acid	77045.7458	175149.3662	33988.83993	253930.7747	310279.1324	220715.3033	513994.445
Spermidine	4350.104934	3112.852502	3619.655038	7941.129058	14018.81391	8585.71126	10476.17242
Petroselinic acid	32548.56439	13364.04294	9155.963495	51038.71043	54799.01416	47012.08639	59788.95214
Sphinganine	970.6805905	3318.484857	288.7612858	5005.44864	5872.37735	5125.363302	11191.22453
Indole-3-carboxylic acid	30.54295684	26.47661199	31.58606372	126.4153412	89.41086694	155.2967294	
Norepinephrine	428058.2564	418224.5344	474951.5454	22580441.94	24379960.72	20402332.37	14876687.57
10E-Heptadecenoic acid	331.4962494	393.9609711	130.553626	371.0931837	161.8176104	255.6532293	
10Z,13Z-Nonadecadienoic acid	242.4619453	273.7773489	240.9095928	306.0583839	159.9989764	218.0958035	
10Z-Heptadecenoic acid	636.714646	775.0424555	251.7114016	755.303301	318.541452	485.1075412	
10Z-Nonadecaenoic acid	29778.62004	85838.4087	9705.985364	113707.3887	57363.51335	83771.22767	
10Z-Pentadecenoic Acid	22373.598	13305.02998	20537.61747	27399.82502	22373.598	26556.35223	
11Z,14Z,17Z-Eicosatrienoic Acid	1895.934752	1781.57269	576.7216601	1787.734446	604.1073941	1162.051998	
11Z-Eicosenoic Acid	34525.72396	702124.0211	10813.0591	717583.7972	409152.2474	435414.253	
12-hydroxystearic acid	27043.23029	5504.187817	16603.60748	31885.21214	26217.35333	33613.55364	31323.94284
1,3-Diaminopropane	297.3872866	238.9738535	249.8233577	214.392241	254.2867419	207.8240394	
13Z,16Z-Docosadienoic Acid	7055.787993	35597.77331	593.6641069	37202.93917	21815.41542	29673.67588	84829.06755
1,4-Dihydronicotinamide adenine dinucleotide (NADH)	562426.6719	282614.1745	183767.4997	308509.0148	90118.57744	276849.1319	155140.0438
1-Methyladenosine	294.2515183	441.9022319	221.8707342	300.348639	392.7798211	219.441301	
1-Methylhistidine	1517.097816	1424.692769	1493.446743	1426.889846	1442.265618	1493.530933	1184.119802
1-Oleylglycerol	621071.4338	192103.7114	759858.8216	1083966.555	561383.9296	1062813.669	1188005.876
2-Aminoisobutyric acid	22.61709375	20.21683386	23.73760535	20.21683386	20.21683386	20.21683386	
2-Aminooctanoic acid	20277.0277	25782.75639	25782.75639	20277.0277	19909.39732	25782.75639	
2'-Deoxyadenosine 5'-triphosphate(dATP)	809.7573458	716.9513754	724.1544129	688.4473913	687.4427705	724.1544129	714.7739617
2'-Deoxyguanosine 5'-diphosphate（dGDP）	20212.70314	18886.10306	20575.01988	23980.74421	37394.0666	22353.78179	35817.21254
2'-Deoxyguanosine 5'-triphosphate（dGTP）	4889.445552	4657.30065	4580.827733	4791.68773	5937.502924	5976.311216	4613.4508
2-Hydroxy-2-methylbutyric acid	14630.28165	14054.29978	8420.733088	19696.96611	9687.058457	21177.72256	1412.75494
2-Hydroxy-3-methylbutyric acid	14179.95154	14430.22842	8069.445671	17702.43813	8816.02044	19892.34846	1555.37785
2-Hydroxy-4-(methylthio)butanoic acid	1254.257313	1061.63494	791.5016185	1284.134649	630.0651064	1228.905744	328.1273703
2-Hydroxyadenine	18.5949135	31.6044978	21.60466388	21.50384698	44.03060409	24.32180807	
2-Hydroxycaproic acid	4358.301241	2833.152844	2349.296054	5197.786259	2947.401065	6788.455881	1177.166722
2-Hydroxypyridine	33.18525098	38.45773394	66.41626575	124.0378729	71.69899972	69.75001036	58.44345616
2-Methylcitric Acid	1542.714111	1138.396285	1080.817687	761.2342027	273.269727	1044.83027	1190.000857
2'-O-methyladenosine	117.0078606	77.9355354	46.28941338	46.02152647	29.72497395	27.51896755	
2-Phosphoglyceric acid	79087.35527	58750.78572	62688.62663	60759.42487	122869.0292	38024.24672	100941.8997
3-Hydroxybutyric acid	9560.777365	15841.94899	10437.39802	12156.93401	11771.52186	14689.98663	7437.226169
3-Methoxytyramine	36.49301142	34.06791092	40.83333857	50.73664566	50.73664566	50.73664566	
3-Methyl-2-oxobutanoic acid	56665.52869	107949.8533	190689.5258	73425.43101	179526.4682	121500.2123	
3-Methyl-2-oxovaleric acid	33740.36666	80555.09421	114380.7939	74839.70858	143559.1045	124587.1955	17029.71796
3-Methyladipic acid	356.0583827	241.168596	250.3377567	319.6676014	258.2041642	299.7460778	
3-Methylhistamine	100.0067028	87.39516859	87.39516859	80.25232211	83.53802984	87.39516859	
3-Phosphoglycerate	76524.3212	59208.96623	59958.49241	56386.6092	123950.7273	33705.39769	110997.5391
4-Guanidinobutanoic acid	9252.041714	7210.822096	8318.095725	7583.334952	6283.241044	7211.534576	
4-Hydroxybenzaldehyde	223.2312726	238.5532626	231.1481532	244.1172625	291.6118895	256.5784755	
4-Trimethylammoniobutanoic acid	2318.740165	1753.385272	2092.608902	1767.897665	1463.711938	1830.014455	2732.798014
4Z,7Z,10Z,13Z,16Z,19Z-Docosahexaenoic Acid (DHA)	7675.015615	988.7541906	3269.144771	7864.339153	3222.025478	3099.155319	18616.54292
4Z,7Z,10Z,13Z,16Z-Docosapentaenoic Acid	200893.8385	198894.7589	61585.30613	182400.4643	64484.02984	117664.4842	486466.0357
5,6-Dimethylbenzimidazole	0.36401394	0.386847952	0.389921848	0.350971431	0.398866636	0.421750924	
5-Aminolevulinic acid	996.2615108	865.8622336	1115.027548	871.0748591	866.3772096	811.1708374	
5'-Deoxyadenosine	1.927346548	2.21794281	2.342451725	2.197980122	2.482580305	2.466559784	
5-Hydroxyindole-3-acetic Acid	97870.09635	77873.95652	51516.10871	107666.6367	47705.12756	77873.95652	131905.6234
5-Hydroxylysine	306.5988273	403.2408095	432.5192644	336.9477118	377.3388168	409.5934337	380.9847447
5Z,8Z,11Z,14Z,17Z-Eicosapentaenoic Acid	87.95087503	2160.779067	1091.723852	1844.213003	804.4521157	1255.039574	3740.339039
6-Phosphogluconic acid	17448.95478	16042.74075	16042.74075	15937.71745	25025.62977	16042.74075	21453.82703
7Z,10Z,13Z,16Z-Docosatetraenoic acid	6595.479431	6398.761259	2247.213726	6811.201961	3331.041558	5033.006962	
Acetylcarnitine	7.551710768	10.94163099	8.536512342	9.266062499	7.096364222	13.1803179	
Adenosine	566.0980757	196.0221026	289.3120642	1857.937117	525.5118097	1077.73646	835.0538689
Adenosine-5'-diphosphate(ADP)	13727.15158	15218.96639	21714.90169	18778.7079	32885.02578	17855.57846	34488.29071
Adenosine-5'-triphosphate(ATP)	13240.46695	14225.15569	13224.9057	12938.08674	14385.73039	14225.15569	
Adenosine diphosphate ribose(ADP-ribose)	74722.19918	77828.66951	179848.6691	200057.1741	320771.6491	200095.2915	78613.63245
Adipic acid	292.0725113	549.0139226	504.4996892	591.5365371	435.8030171	75.85663606	
Alanine	843437.0109	809065.1924	997851.2832	812158.2879	779655.8287	885956.4288	541910.4686

Supplementary material 5

Alanylglutamine	930.7620986	379.7962574	251.8958508	614.7226509	222.3759278	1159.186694	491.1324684
Allantoin	72736.53357	72733.09093	65385.81826	47335.77032	64212.44829	57289.35789	50467.19091
Alpha-aminobutyric acid	20.64095068	25.80708748	25.41980594	24.04115119	25.51425947	24.09026985	
Alpha-Linolenic Acid	283.5369035	304.4344864	209.5854901	248.7879431	197.1167392	238.2733072	
Aminoadipic acid	36186.07049	34505.37576	36012.56623	34312.29643	32986.73986	34877.58142	44323.48614
Aminocaproic acid	56435.22033	48840.54494	61317.86539	58142.14615	53230.1325	60047.12983	47716.4319
Anandamide	106.0327799	39.31321201	64.74482157	92.77931582	76.64129772	60.51532067	
Arachidonic acid	50061.49155	41184.63599	20660.6878	51586.92973	30020.29675	40497.86368	80817.37806
Arginine	138973.5653	121831.1551	123137.8884	112019.2481	99468.54697	128858.6821	106516.8933
Argininosuccinic acid	9866.599265	8373.491862	8576.749552	5733.458222	7913.428091	4036.500628	5059.997493
Asparagine	194631.7421	178182.6256	181042.3607	206035.0086	161370.1622	210938.3913	130749.6207
Aspartylphenylalanine	2062.028469	1769.536787	1957.141638	2267.977883	1604.601091	2893.086893	2675.942139
Asymmetric dimethylarginine	16152.70792	11027.59806	12893.87235	10142.76483	12001.76682	12003.19804	14789.4702
Azelaic acid	5464.657028	3630.463413	2750.804859	8022.595937	3633.095629	6520.749908	4402.318321
Benzamide	39.90019726	51.70869621	54.01854567	39.84437027	39.33767137	36.511581	
Benzoic acid	1659.197261	878.6788789	1061.075631	1410.60341	2191.593815	1642.454267	
Beta-Alanine	882528.4573	809865.5356	1021139.221	812306.7622	791243.1621	888753.2102	565190.6661
Beta-Glycerophosphoric acid	1828751.53	979891.467	898354.5981	574817.7632	291052.4643	207166.1315	203008.8791
Betaine	5622.379646	7008.755582	6313.008789	5733.725201	4579.95979	5282.672814	6752.75529
Biliverdin	27474.58771	22863.50475	20298.80635	23250.93403	42081.08865	25599.7532	35645.09879
Caffeine	7.184732153	5.529431274	4.075972677	6.5815942	5.488478104	7.089641518	
Carnitine	4029.262399	3384.974121	3810.117123	3547.501732	3219.341624	3881.440904	8410.930923
Carnosine	915.2424636	935.8398995	107.4740451	722.3215127	753.2629369	1189.340616	
CDP-Ethanolamine	13170.7658	8418.406763	14296.63858	28736.50237	9082.86206	36762.41476	39675.17577
Cellobiose	176324.3296	192137.1385	72502.69632	101532.9817	27393.45358	20833.07914	
Cholesterol sulfate	166.3176583	811.64102	136.0814999	364.4029579	353.339925	280.4615487	
Choline	866.3106643	570.1306038	770.4196867	794.2114728	799.6414173	1231.755783	1789.006621
cis-4-Hydroxy-D-proline	23518.9241	21639.72351	27195.12368	24443.50331	23033.61259	26709.67514	20903.89212
cis-Aconitic acid	10968.93007	9435.850629	7376.541732	6892.879053	10227.47623	9643.111299	22332.92464
Citicoline	49033.4332	43723.16632	55185.44403	53916.12312	48179.78273	54107.92043	82959.86834
Citric acid	287372.2566	248740.0835	250583.705	254074.4091	314436.6599	217974.1733	364875.6872
Citrulline	6791.380505	4352.752995	4037.103532	5557.978199	3210.933706	5400.112813	3967.199057
Conjugated linoleic acids (CLA)	4551.172683	4664.998407	2719.867405	4815.279931	3109.527136	3988.44738	
Cortisol	19.3842478	13.86199018	19.02125058	122.8453964	28.26456243	170.5953821	
Cortisone	76.31833589	96.74184695	96.74184695	76.31833589	59.91462346	96.74184695	
Creatine	582967.3418	514828.2292	607198.8479	628841.7279	649528.4044	616227.3213	470406.7999
Creatinine	18264.55129	11087.72018	7431.239255	19058.44548	9216.012745	12449.37402	15781.03206
Cystathionine	6712.702075	15812.87132	9715.040781	12042.91606	15738.74861	22126.36948	5423.177732
Cystine	32556.24185	16540.91142	9915.892142	27725.43529	12949.66389	26503.76833	23377.89484
Cytidine	235.0834565	284.2619904	334.3804121	247.8801503	423.730539	153.3952034	
Cytidine 5'-diphosphate（CDP）	4627.074216	6061.48698	5781.317556	4701.521272	6888.454114	4631.346284	6685.876678
Cytidine monophosphate（CMP)	27690.47092	26056.19029	36476.06145	37038.9047	35057.16105	36080.04657	85974.18587
D-2-Hydroxyglutaric acid	212079.8666	160999.2514	192620.8127	172783.4801	139129.2573	143218.8106	274582.4444
Decanoylcarnitine	0.939421913	0.833996026	1.04383204	1.042231726	1.639282293	1.57009604	
Deoxyadenosine	105.4817953	74.88434225	60.17055773	96.04611769	63.38543575	75.86818521	
Deoxycholic acid (DCA)	369.4292521	263.5617093	350.7575552	395.6968323	586.6573395	457.6641699	
Deoxycytidine-diphosphate	6494.320901	6200.756714	6841.04223	6495.491196	6695.343188	6316.846723	8124.959815
Deoxyguanosine	16106.69884	16036.41382	16121.99224	15997.62385	16169.88976	15981.24946	16497.40993
Dephosphocoenzyme A (Dephospho-CoA)	1259.155987	1006.885584	815.7770428	1278.627302	878.5593819	961.4666847	1307.000112
D-glucosamine 1-phosphate	1982.072102	1483.263172	1431.513624	1075.902639	4476.116755	1483.263172	
Diethanolamine	5364.166824	13708.07364	10462.12357	9068.050141	6529.370828	4693.01782	
Dihomo-gamma-linolenic acid	2015.809637	1818.127021	622.2621174	1960.221184	635.4649993	1212.537524	
Dihydroxyacetone phosphate	718.001137	578.0003769	820.0337375	730.5694452	273.3801128	845.190461	
Dodecanoylcarnitine	2.225279125	1.699694431	2.247301955	2.208422605	3.355246421	3.275360695	
Dopamine	57.35054252	76.75055564	56.56313574	68.03045236	78.79926085	79.42814693	
D-Ribose 5-phosphate	125565775	101736796.5	113336264.1	117749216.5	193692600.4	112752084.6	274922228.4
Elaidic Acid	19950.3379	6764.305215	5965.364111	24898.30949	7754.17916	16134.34634	46296.17721
Epinephrine	134.7101177	168.4066671	33.77981164	34.36029972	42.63582656	35.5945733	
Erucic Acid	13395.06469	62874.88589	61392.67087	187093.6621	100353.6147	69468.56294	475429.0624
Erythronic acid	2372.036145	2005.319538	2365.623708	2339.859464	2003.311826	1878.090523	2617.578885
Ethylmalonic acid	983.8500886	1066.547068	983.7972562	1000.471052	759.993751	870.3905102	
Flavin adenine dinucleotide(FAD)	23481.94683	22965.94176	23759.55754	22399.05094	21816.28254	23759.55754	42362.44744
Fructose 6-phosphate	156567.5052	175130.1407	177229.805	157416.4947	946806.4683	130359.5658	788974.9558
Galactose 1-phosphate	14701.23253	15309.74897	18292.35115	15105.68409	56126.79721	15501.71874	77990.81592
Galacturonic acid	37549.06978	33741.11414	36358.1938	32631.46412	29251.03645	21421.74934	70288.88919
Gallic acid	4695.531956	3843.79648	4488.338649	6935.897621	6935.897621	6935.897621	
Gama-glutamylalanine	26925.39391	15163.16089	7796.516695	21881.68495	9252.975456	20845.36492	17687.83468
Gamma-Aminobutyric acid	14630.33796	14233.99702	11732.80371	13130.0237	10913.75647	12690.89667	2688.52452
gamma-Glutamyl-methionine	15970.06988	9318.343339	4469.354864	11846.29749	5897.735486	13311.97868	11142.96807
gamma-Glutamyl-phenylalanine	16407.6767	9562.198348	3241.669879	9928.786014	2889.081591	8698.929109	6947.326465
gamma-Glutamyl-valine	22165.83488	8841.861682	3662.003804	16287.84935	4183.887184	14084.04362	12913.92902
Gamma-Linolenic acid	120.1892323	129.9239344	86.71661124	105.4281565	81.70515642	100.7170691	
GDP-fucose	99622.21751	76342.57529	81446.47378	87300.73745	67292.48829	73196.64509	93462.64376
Gluconic acid	45943.44522	43011.94029	39193.30592	38747.42023	34233.17275	36287.02543	33217.74931
Glucosamine 6-phosphate	1719.954398	1211.0496	1764.787196	1666.706878	3991.598259	1376.823417	

Supplementary material 5

Glucose 1-phosphate	17069.93038	14500.01421	16743.7646	15173.18243	63277.86802	11891.53487	81614.17526
Glucose 6-phosphate （G6P）	49155.87831	38355.68212	49279.53568	44354.26087	276404.7223	38596.04912	375331.3642
Glutamic acid	2084567.924	1779197.499	2042379.596	1995023.625	1827483.237	2002605.585	2508960.208
Glutamine	902026.1299	864225.9186	1159305.247	717034.4729	954323.2432	957082.8623	1832249.762
Glutamyltryptophan	193.3196986	96.60018341	63.35576531	210.8083223	88.02726468	195.1441846	
Glutarylcarnitine	229.6277916	223.1433434	177.2405371	215.2332771	180.363407	221.8422754	349.7417445
Glycerol 3-phosphate	1048387.782	538221.8735	463249.2626	325761.0304	218422.3093	98181.70104	96748.95858
Glycocholic acid （GCA）	76.38536763	74.26564467	87.65093844	68.01759083	66.24542009	84.63892396	
Glycodeoxycholic acid (GDCA)	103.8459552	102.3541999	154.4738839	104.998263	88.73926695	138.5276558	
Glycolithocholic acid-3-Sulfate	160.5877895	150.0270712	213.8029099	189.3585271	257.5402865	217.921987	
Glycyl-glycine	8034.260752	6044.702266	7148.352428	7535.393804	5803.077344	7643.445205	6111.506398
Glycylleucine	27778.06095	13297.00733	10597.13162	17683.00547	7946.810903	13356.64334	20921.36624
Glycyl-Phenylalanine	737.3320516	380.2804817	270.2662982	678.0938481	308.0804034	558.3945586	544.6325024
Glycylvaline	72676.21492	43213.35564	30433.227	54956.44658	34447.50796	46069.67051	54757.85747
Guanine	32902.75032	36995.38693	31898.93057	38315.83983	46442.34547	25384.29899	47109.66544
Guanosine	2125.078721	2658.044444	2570.102164	3247.988246	3382.243372	5910.531979	5560.8219
Guanosine diphosphate(GDP)	26404.66079	30429.98947	29163.88435	31404.56024	39913.80408	29318.5208	32824.98398
Guanosine monophosphate （GMP）	25638.25894	33415.48483	31238.08743	29704.78659	39001.66086	21995.42875	127031.2199
Heptadecanoic acid	21768.88983	41568.56672	8971.223571	48167.50145	53235.05675	46734.59026	63511.57165
Hippuric acid	27.15259797	18.96310648	22.24944701	23.70057717	21.34204351	24.91969526	
Histamine	813.8255925	412.7316866	563.0614905	717.7556971	628.3519673	654.3255062	
Histidine	190111.0614	175300.2008	188501.5544	172901.9145	157995.7481	171336.8626	131228.205
Histidinol	25.61101894	17.02991607	18.68406664	14.41841703	25.05056295	18.68406664	28.06861338
Homoserine	684412.4577	615230.9674	772321.1122	681737.8245	598388.4752	725034.7214	498827.947
Hordenine	25.15217539	23.30736024	26.76312621	26.80412779	25.33215328	26.33484748	
Hydrocinnamic acid	9676.100283	10817.22155	10817.22155	9676.100283	8306.935214	10817.22155	
Hydroxyphenyllactic acid	12373.64778	10163.38608	7922.275203	12641.38862	7137.627499	10450.71176	6286.896279
Hypotaurine	44138.38949	41821.1492	40536.72744	45029.82955	37002.95039	39914.94225	92054.88706
Hypoxanthine	2526060.394	2169313.473	2269237.724	2285014.305	2063262.294	2077889.788	2300909.246
Indole-3-carboxaldehyde	97.18551369	76.168135	94.92497529	59.46918665	93.53464635	194.9127639	216.1972687
Indole-3-lactic Acid	1342.467915	841.9463402	627.8195383	1934.71261	806.7712351	1661.773977	481.2020396
Indoleacetic acid	15.71278597	78.13674742	13.85837132	42.55004299	21.73485743	52.02500323	
Inosine 5'-diphosphate(IDP)	8817.206567	9714.292717	11387.05327	9853.700643	12616.91947	10305.57858	14081.47205
Inosine-monophosphate(IMP)	5922.362443	4126.217122	4385.270984	6937.005896	5334.867641	5028.333288	7441.304552
Inosine triphosphate(ITP)	217.289717	168.4003388	164.1204349	157.3099491	264.1066344	189.6513089	323.5587035
Isocitric acid	1731.537433	1841.635672	1530.229294	1435.813876	945.2469914	1323.20118	
Isodeoxycholic acid (IsoDCA)	547.4004133	405.9805336	512.3915376	567.6917369	38.4300288	747.7988628	
Isovalerylcarnitine	45.59592961	1897.359153	34.39063074	38.20161994	47.18578104	45.438372	713.2261289
Ketoleucine	92.23525578	346.1671757	517.8943798	109.5390097	197.3661702	185.2982319	
Kyotorphin	592.3789509	529.4246231	636.2572253	543.8215743	521.1856459	552.5859581	515.4152317
Lactulose	4017.672093	3968.553715	2023.344064	2463.83403	2334.724428	2023.344064	
Leucylalanine	915.0457795	557.8920391	403.8669142	720.5242836	342.563697	590.7368753	617.9870578
Leucyl-Glycine	65779.70234	27755.17918	38416.12264	40591.97985	20261.68683	26831.25678	32660.73406
Leucylphenylalanine	174.6898844	31.4792545	15.01447926	213.3325791	69.03396498	177.9314136	140.6054887
Linoelaidic acid	4487.771725	6070.301686	2611.559627	4781.944689	2939.513328	3868.227041	
Linoleic acid	6472.143325	8583.387187	3628.24303	6656.639031	4219.87043	5577.109529	
Linoleoyl ethanolamide	28.50571741	9.150745145	20.09409349	29.72712461	25.12774874	25.4092216	
Lumichrome	1274.607445	1038.048242	1400.583463	1483.750291	1381.710469	1524.548354	1761.23534
Lysine	451491.9993	319443.013	381408.283	361951.163	263651.2543	373549.5473	261537.1925
Maltotriose	108025.0883	104912.6312	114462.2812	95935.95768	106395.7766	134400.1997	195951.761
Mannose 6-phosphate	201872.9564	217282.8853	211657.2075	199985.3431	1212373.044	215990.2395	837975.7268
Methionine sulfoxide	2911.59357	2695.056417	2715.13398	2540.479543	2008.09103	3063.396854	1031.499882
Methylglutaric acid	302.6187411	511.20663	462.2253813	6.818929068	436.3340442	129.6045303	
Methylmalonic acid	48911.96664	6271.85925	3691.055193	36518.32747	25817.9136	27494.91052	
myo-Inositol	2140778.214	2050467.104	2301768.008	2357236.699	3257755.513	2704129.306	1857851.259
Myristelaidic acid	471443.0803	472849.1486	332748.8839	470944.1405	381620.5697	408265.511	1442367.424
Myristic acid	2858.869641	1736.074784	2459.46594	2919.659203	1758.865031	2354.131352	
N6-Acetyllysine	198.6947658	105.9860352	107.5905955	121.9768138	82.97705604	150.0222041	105.3235573
N-Acetylalanine	79993.07233	56509.31015	70278.36472	60079.45498	57163.20901	49258.61833	83294.73023
N-Acetylaspartylglutamic acid (NAAG)	233.238897	93.08951465	31.43084774	169.01877	388.8788102	491.3746635	535.0740281
N-Acetylcadaverine	10.48397153	20.54526345	20.54526345	22.26211782	32.15245318	7.259265852	
N-Acetylglutamic acid	20487.41632	16187.84105	16097.2932	14994.87031	11809.00327	14680.43048	14414.65112
N-Acetylleucine	62.61002606	49.60591275	62.08343565	75.41784138	0.057046699	75.84840859	
N-Acetylneuraminic acid	57091.75779	49465.05717	60134.41319	56389.11204	41309.99677	60253.05848	37467.19683
N-Acetylphenylalanine	23.25515125	14.57626952	13.25800845	55.52848591	4.529291615	33.64130877	
N-Acetylputrescine	1035.72978	679.7603763	427.2535195	671.1620812	618.9344794	365.7882449	2152.382158
N-Acetylserotonin	0.213567463	0.25373832	0.25373832	0.213567463	0.133225747	0.25373832	
N-Alpha-acetyllysine	95.73454287	69.7827973	82.46127619	85.81793796	79.55792715	91.12465184	94.78321316
N-Formyl-methionine	652.4617702	416.1213634	538.7898219	421.2986758	389.545014	444.4854341	966.1632133
N-Methylalanine	226.4223272	131.2014763	236.5974003	183.0249475	259.8408514	188.8884436	
N-Methyl-aspartic acid	16366.43397	16154.61463	16178.33998	16112.55912	15986.07813	16241.31926	16187.01049
Norvaline	342956.29	284813.4175	343541.2657	274083.0123	286960.7221	298198.6698	197047.1082
Oleic acid	24287.17324	17624.4757	8152.698414	29821.69545	14636.52355	21703.61912	52011.99422
Oleoylethanolamide	4.177848737	0.9642445	3.429357352	3.151358462	2.264576422	3.150279756	
O-Phosphoethanolamine	12034.06184	6429.264557	7698.02425	15495.44119	27877.55874	12256.15349	139667.6353

Supplementary material 5

Ophthalmic acid	2536.402854	2267.298759	2165.885173	2446.361109	2138.757599	2234.216621	3155.991741
Ornithine	8487.617314	7798.913104	8461.327741	8112.208548	9673.666425	10271.53176	8041.677101
Orotic acid	26033.36527	26923.08424	29540.28566	40187.83926	32113.83803	79118.00801	22151.53786
O-Succinylhomoserine	2641.872705	2237.624368	2104.841113	2124.897703	2262.921112	2566.016594	2000.910603
Oxidized glutathione	197420.0577	150873.897	180656.7976	157752.1219	121831.7375	147496.3718	308845.182
Palmitelaidic acid	7360.814654	5769.409227	4136.414898	7434.941924	3919.453463	5265.686274	
Palmitic acid	53557.70581	56841.19608	29576.37895	75595.98444	42013.02237	53600.32962	169698.7121
Palmitoleic Acid	12693.33306	10994.78835	5773.957364	12292.35147	6052.052407	8390.332408	
Palmitoylcarnitine	235.9634338	147.8235826	185.7423078	227.9966097	229.9251989	224.9472345	907.2984253
Palmitoylethanolamide	43.98619433	39.83497562	39.79223669	36.15525802	38.35552287	37.21458786	262.3925389
p-Aminobenzoic acid	39980.68857	33766.76015	28409.57236	28409.57236	29401.4674	36120.4907	
p-Cresyl sulfate	4226.977718	2580.989925	1613.898521	5938.33862	2221.927149	4951.664312	2720.28597
Phenylacetylglycine	1012.372029	774.8888905	859.5632857	850.8928895	726.8506159	890.0454751	450.8882543
Phenylalanine	4096189.068	3092059.361	4032584.392	3278976.313	3415005.834	3466129.867	3134109
Phenylalanylalanine	147.7592886	108.6100135	94.26198665	125.0280899	96.04906746	122.5603292	142.097123
Phenyllactic acid	6968.446805	5334.289707	1900.565858	8204.202832	2720.985258	7033.263435	1927.272814
Phenylpyruvic acid	1795.819734	2907.403786	3974.202518	2126.585695	5856.015073	3998.411209	1566.452301
Phosphoenolpyruvic acid	1328654.268	761355.8726	981261.5551	812009.9881	926645.4605	494257.5087	1170375.405
Phosphonoacetate	41998.25486	33715.96047	34999.27164	39144.98904	48911.3169	32554.17038	59114.28855
Phosphorylcholine	125809.4057	111601.0274	107623.1351	113115.1164	112828.2882	96850.21335	291650.489
Phosphoserine	2732.732117	1800.311237	1920.405427	1850.184159	2704.563739	2101.579845	5359.785845
Pipecolic acid	1563.641916	969.3870313	1662.248316	1239.372205	1613.51357	1341.892189	1327.143842
Proline	2412.360987	1424.990684	2290.509229	2400.379954	2725.066745	2580.408692	1653.935974
Propionylcarnitine	4880.786848	6114.476612	5701.01145	5628.247388	4105.089502	5043.483467	19203.30481
Purine	97.18061156	97.37396335	97.37396335	97.07834753	95.96304056	42.81693462	
Putrescine	22864.62079	16355.31592	17842.40917	23872.50497	19166.41842	18921.21996	39573.12394
Pyridoxamine	2846.82373	2499.189276	1643.50772	3688.780809	1814.664485	2754.672169	2072.521865
Pyroglutamic acid	1438.347021	937.153398	1443.011245	1133.046059	1459.734322	1237.356546	931.0218703
Pyruvic acid	96419.34059	71167.01872	96099.25529	74667.92425	70413.87554	74759.49529	
Quinaldic acid	15.93756722	19.48720237	21.06551523	14.11745935	11.96323914	18.84299095	20.76496642
Retinoic acid	27.80599847	27.3425228	21.99510562	31.95650143	26.53416493	26.31797853	
Ribonic acid	198.3023211	183.8988202	280.3731926	717.0221716	244.2851072	584.3347071	
S-Adenosylhomocysteine	30162.0491	25348.97824	33092.47823	29182.24405	25267.93994	31685.42468	26674.06148
Salicylamide	143.8959697	120.6197263	125.2577991	143.7971192	152.0354775	133.7034162	
Salsolinol	82.73351611	61.73098008	76.5873036	81.4503509	74.16750996	68.45621804	
Sarcosine	806066.1882	743015.3884	954788.8918	761332.7436	699816.9624	838256.5558	501273.6983
Sebacic acid	1192.270643	616.2675309	428.1630139	1262.357781	760.5742813	1319.195378	868.0334673
Sepiapterin	322453.1345	285091.8101	406285.1362	351085.1264	412742.8945	331839.6147	333081.0327
Serine	312975.3019	261913.7925	273246.9251	300001.3941	238476.9794	323981.3864	300498.6629
Stearylcarnitine	362.8555828	584.0272097	264.736386	548.9643966	597.9921523	560.5994707	5291.583734
Suberic acid	25.365167	20.87101278	19.66399523	27.94496832	24.47471548	29.4231359	
Sucrose	84138.02403	123767.6261	3536.620967	57665.19021	8168.819865	3235.509212	
Taurine	3539.832321	3810.160281	5734.682223	4150.792451	4615.741409	4871.110175	5051.099435
Taurochenodeoxycholic acid (TCDCA)	63.22848063	81.61753078	47.2911673	35.15234661	6.003325545	76.33305353	
Taurodeoxycholic acid (TDCA)	227.8153377	243.6642293	236.3803867	200.1053965	170.4949703	252.6749113	
Taurolithocholic acid (TLCA)	4.225571128	3.339984181	3.453231314	4.001965699	3.284083127	3.283310265	
Tetradecanoylcarnitine	196.0815068	154.5114965	175.4666944	133.0951909	177.4048511	187.9804468	374.1016468
Thiamine monophosphate	5216.670665	5178.218597	5198.787687	5187.609646	5226.578105	5199.155912	5357.139935
Threonic acid	2040.835277	2033.591607	2225.42896	2171.194888	1807.850184	1891.763836	2740.245622
Threonine	538383.5853	480461.2061	613057.9146	525575.86	474649.1708	551415.0074	391353.9548
Thymidine	1055.897451	755.7524604	1042.877294	870.5493507	1008.78931	690.4414739	
Thymine	1167.495562	791.5780301	1145.74205	915.0895092	973.2509584	1007.208612	
trans-Aconitic acid	17558.00372	15752.45999	13663.86847	12957.31588	17148.74292	15233.36278	
Trimethylamine	3184.637744	2401.805804	2931.627649	3114.947352	3436.12896	4032.298253	
Trimethyllysine	1467.038826	5249.163769	5559.989962	5599.857981	4329.208714	5357.794127	
Tryptophanamide	71.5255107	90.91162989	92.77560102	53.74003783	47.94422848	72.12452873	
Tryptophanol	88.29945207	104.1201348	17.4397969	88.29945207	68.73831103	104.1201348	
Tyrosine	496332.6915	411087.7032	500912.9551	401541.9892	402506.9802	444568.5769	322429.8331
Uracil	234857.2214	151215.7391	176972.1638	161584.1458	144574.4731	140233.464	230925.2648
Ureidopropionic acid	132644.657	142309.8543	139773.3736	148291.7305	128654.4021	142810.9179	131952.6387
Ureidosuccinic acid	48435.09986	27686.02766	22489.57187	20244.35215	12137.24114	13984.48233	19216.24475
Uric acid	1157.859627	1906.303614	1691.628464	1414.37132	1317.826095	1593.513669	1527.309329
Uridine	13785.0214	5525.145363	7531.621918	8989.521557	9258.358255	5739.932133	21679.34538
Uridine 5'-diphosphate(UDP)	137622.9918	134205.4817	85208.49303	113544.6595	86582.74312	85437.80916	264788.1106
Uridine 5'-monophosphate(UMP)	18199.37296	15716.07229	19921.05965	18829.92391	19760.51525	14423.88169	48043.70431
Uridine diphosphategalactose(UDP-galactose)	96616.4128	49064.68509	68449.10643	45474.31679	53565.56615	49505.31941	259957.2299
Uridine diphosphate glucose(UDP-glucose)	118030.6389	68732.66424	134203.9635	75559.96522	94803.44842	75966.27067	346611.5964
Uridine diphosphate glucuronic acid	84382.08142	79445.30971	73261.84968	74312.61017	106123.1461	82709.75597	170436.8265
Uridine diphosphate-N-acetylgalactosamine	154400.1915	148690.406	208127.1315	148033.7651	259104.7547	264467.5168	384754.6875
Uridine diphosphate-N-acetylglucosamine	110546.4613	117494.6814	104757.3164	94091.4989	147784.4409	133701.4923	274743.7543
Valerylcarnitine	15.23481421	3304.75329	79.94718985	82.68721815	16.8008913	9.447618226	1160.13984
Valine	384831.0625	305044.6319	375581.9033	303311.9265	324130.7378	341062.1444	212888.049
Valylalanine	16630.79555	11129.18748	8203.558805	11713.94965	6256.839158	9913.764845	13105.06825
Vanillic acid	33.64118778	23.7274543	33.35505125	31.65286616	30.99408936	38.3049371	
Vitamin B1	30846.07141	1505.377653	26854.48371	20364.526	17322.11839	19016.90922	29716.7516

Supplementary material 5

Vitamin B9	3004.907778	4715.731229	3300.362603	1699.718978	3529.757508	2413.182547	789.1537837
Xanthine	277636.0191	113650.3822	209981.7133	130104.8383	337948.3066	294372.2809	98299.2957
Xanthosine	39.58794684	521.7541725	859.9432102	821.0538255	518.4912226	741.2926257	
Xanthosine 5'-phosphate(XMP)	1679.2576	2890.754089	3226.615301	3271.246624	4689.825345	2073.411735	1106.639346
α-Ketoglutaric acid	393418.9918	612113.8507	783430.1704	492389.8347	751972.5511	544854.5916	604563.2136
β-Nicotinamide adenine dinucleotide (NAD)	472866.3694	542680.6037	982993.0972	338920.3237	893815.187	488894.1287	418026.4582
β-Nicotinamide adenine dinucleotide phosphate (NADP)	631.7389056	194.238788	478.9148722	1005.26802	623.5670024	592.7736162	

Supplementary material 6

Fig1 F

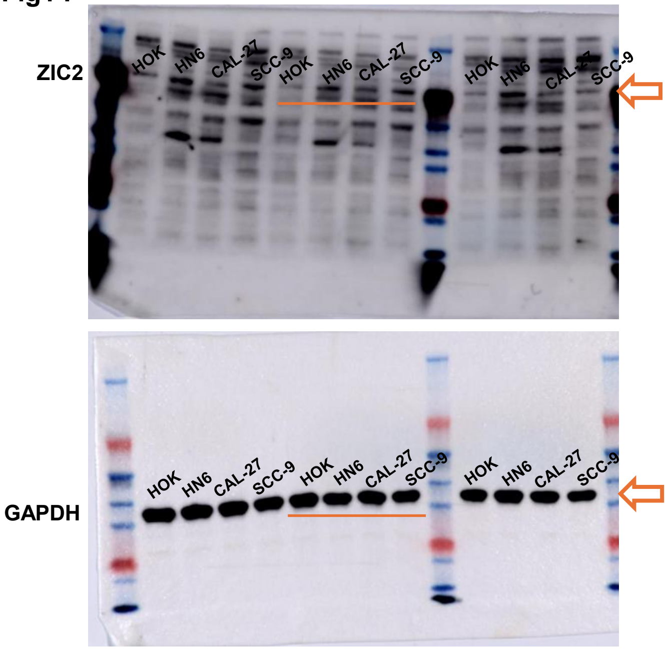

Paracancer tissue=P

Cancerous tissue=C

Fig1 G

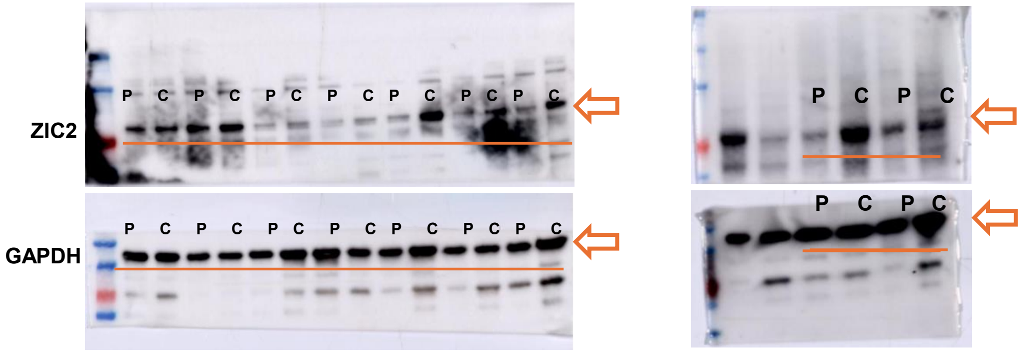

Supplementary material 6

Fig2 C

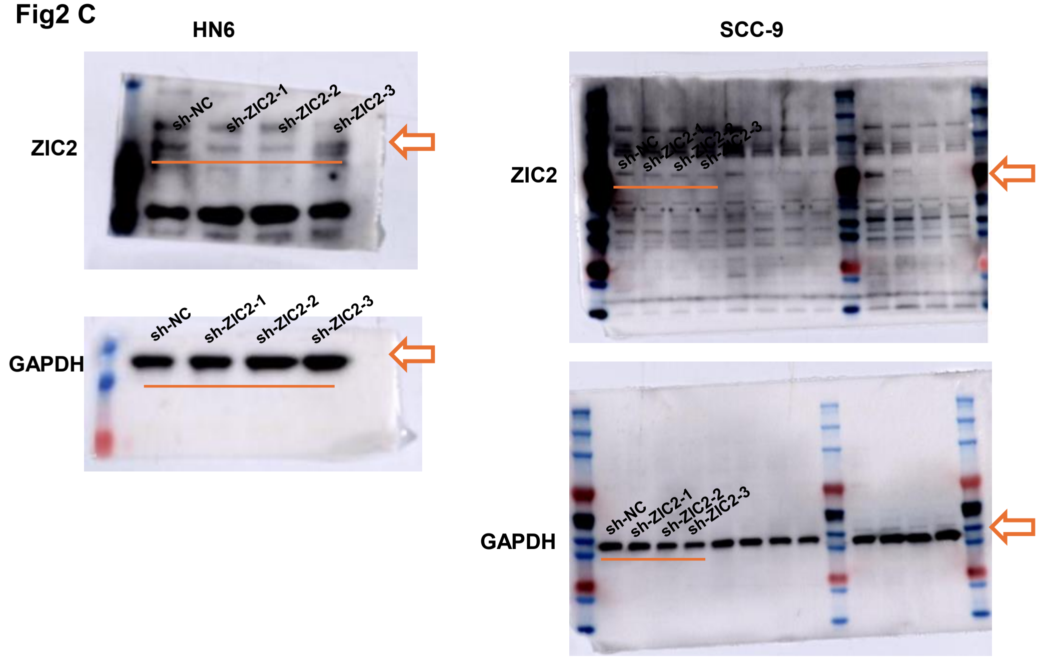

Fig3 B

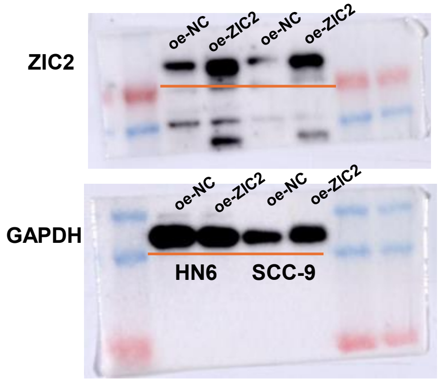

Supplementary material 6

Fig5 C

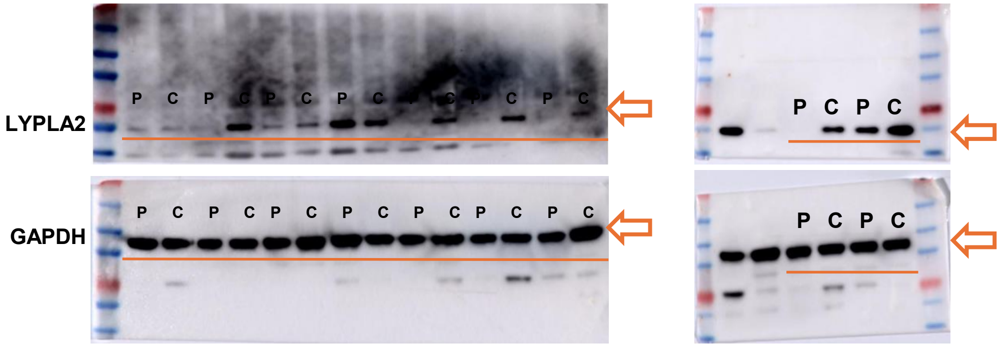

Fig5 F

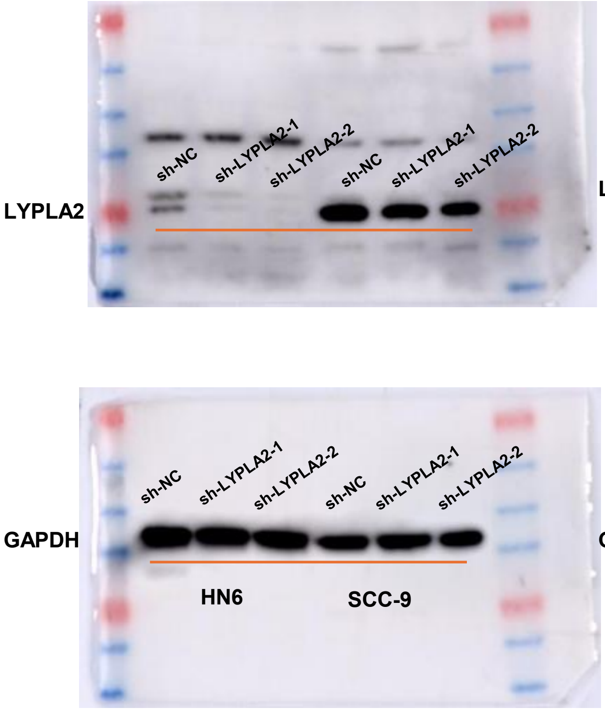

Fig5 G

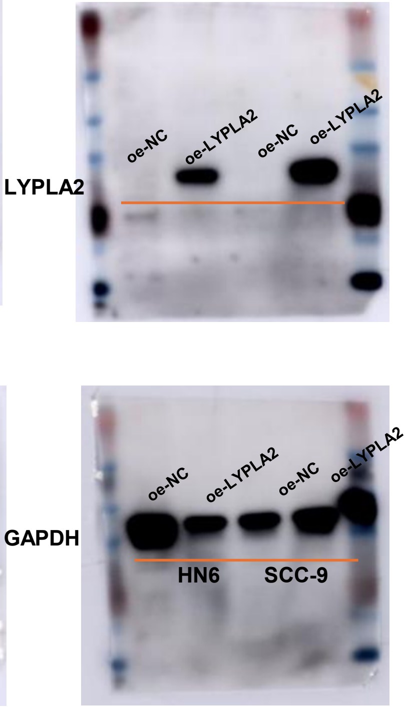

Supplementary material 6

Fig6 C

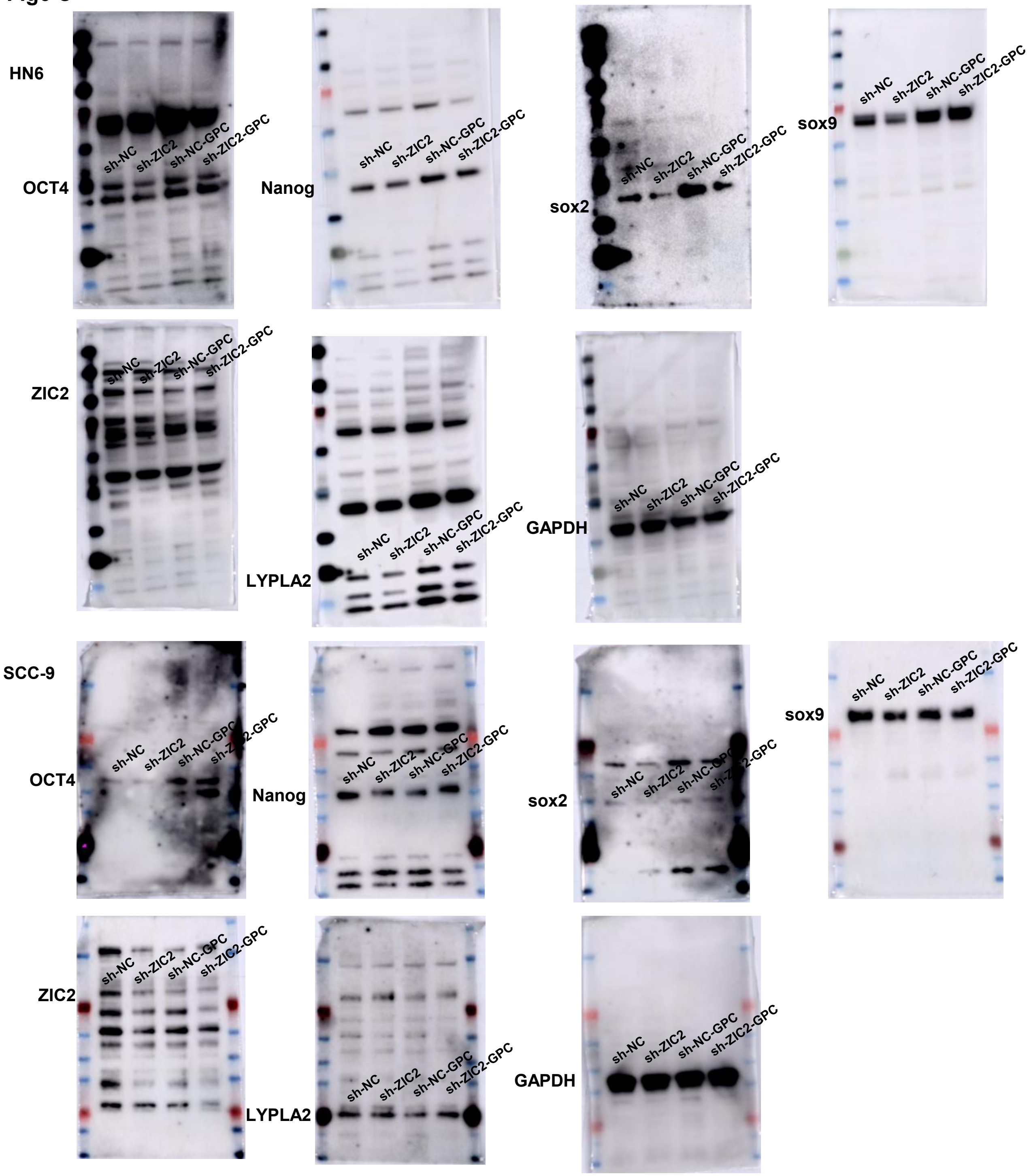

Supplementary material 6

Fig6 G

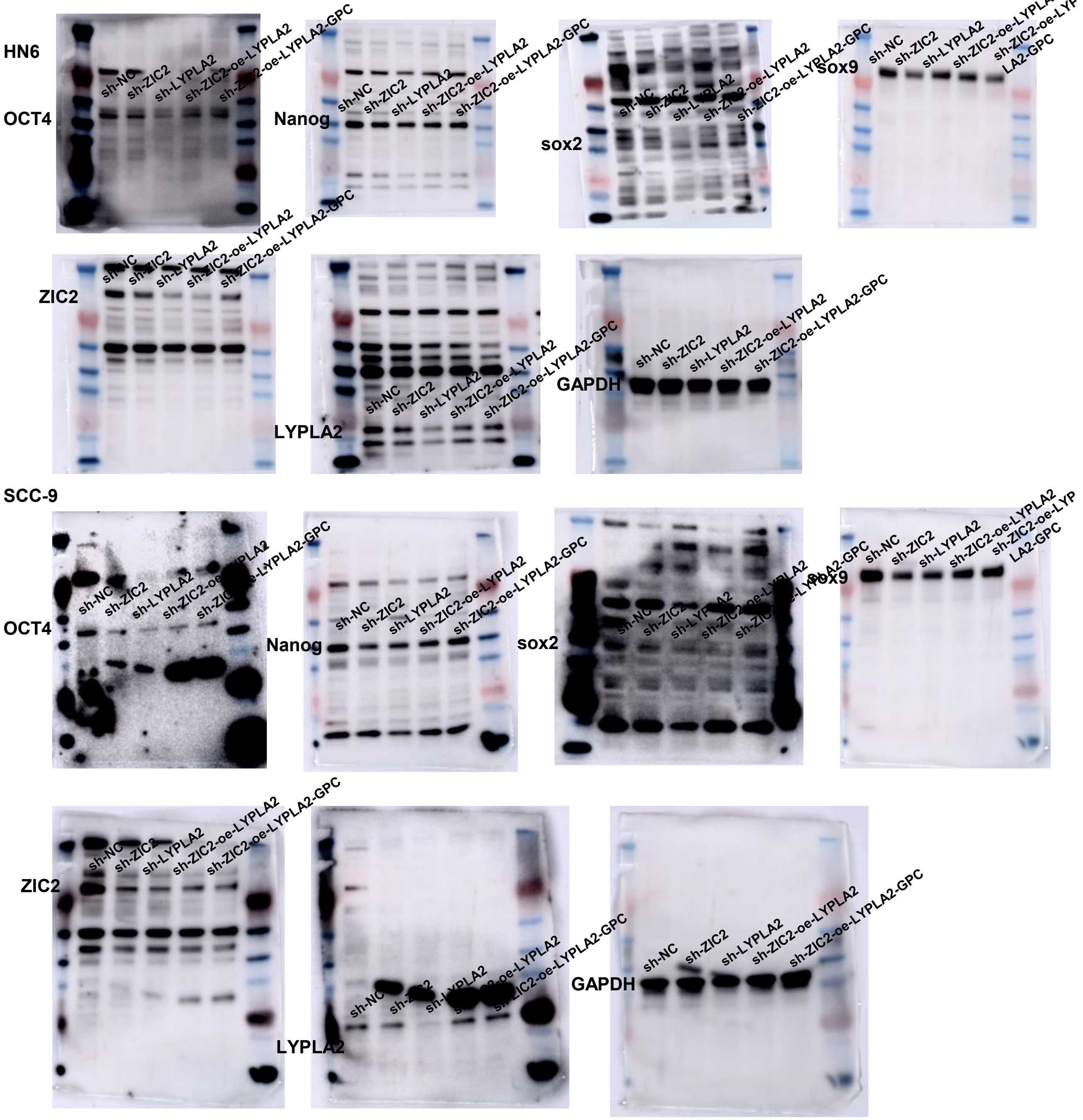

Supplement: Supplementary file 1 — Supplementary material [file 41419_2026_8483_MOESM1_ESM.pdf]
